# Aptamer-drug conjugates-loaded bacteria for pancreatic cancer synergistic therapy

**DOI:** 10.1038/s41392-024-01973-3

**Published:** 2024-10-14

**Authors:** Yu Xiao, Tao Pan, Wuren Da, Yuanding Liu, Shuangya Chen, Daiquan Chen, Keying Liu, Yihan Zheng, Daolong Xie, Yuan Gao, Haiyan Xu, Yang Sun, Weihong Tan

**Affiliations:** 1 Institute of Molecular Medicine (IMM), State Key Laboratory of Oncogenes and Related Genes, Shanghai Cancer Institute, Department of Oncology, Renji Hospital, School of Medicine, Shanghai Jiao Tong University, Shanghai, China; 2 Zhejiang Cancer Hospital, Hangzhou Institute of Medicine (HIM), Chinese Academy of Sciences, Hangzhou, China

**Keywords:** Drug delivery, Drug development

## Abstract

Pancreatic cancer is one of the most malignant tumors with the highest mortality rates, and it currently lacks effective drugs. Aptamer-drug conjugates (ApDC), as a form of nucleic acid drug, show great potential in cancer therapy. However, the instability of nucleic acid-based drugs in vivo and the avascularity of pancreatic cancer with dense stroma have limited their application. Fortunately, VNP20009, a genetically modified strain of *Salmonella typhimurium*, which has a preference for anaerobic environments, but is toxic and lacks specificity, can potentially serve as a delivery vehicle for ApDC. Here, we propose a synergistic therapy approach that combines the penetrative capability of bacteria with the targeting and toxic effects of ApDC by conjugating ApDC to VNP20009 through straightforward, one-step click chemistry. With this strategy, bacteria specifically target pancreatic cancer through anaerobic chemotaxis and subsequently adhere to tumor cells driven by the aptamer’s specific binding. Results indicate that this method prolongs the serum stability of ApDC up to 48 h and resulted in increased drug concentration at tumor sites compared to the free drugs group. Moreover, the aptamer’s targeted binding to cancer cells tripled bacterial colonization at the tumor site, leading to increased death of tumor cells and T cell infiltration. Notably, by integrating chemotherapy and immunotherapy, the effectiveness of the treatment is significantly enhanced, showing consistent results across various animal models. Overall, this strategy takes advantage of bacteria and ApDC and thus presents an effective synergistic strategy for pancreatic cancer treatment.

## Introduction

Pancreatic cancer, predominantly pancreatic ductal adenocarcinoma (PDAC), is characterized by a high mortality rate. Notably, 80–85% of patients are diagnosed at advanced stages or have already experienced metastasis, eliminating the possibility of surgery.^1^ The primary clinical treatment for unresectable, metastatic advanced pancreatic cancer is chemotherapy.^2^ However, chemotherapy carries significant toxicity and has limited efficacy in improving patient survival. In a phase III clinical trial of advanced pancreatic cancer patients receiving FOLFIRINOX treatment, the median overall survival for the FOLFIRINOX group was 11.1 months,^3^ while the gemcitabine group had a median survival of 6.8 months.^4^ In recent years, we have witnessed continuous research endeavors aimed at developing novel treatment strategies and combinations to improve outcomes for pancreatic cancer patients. These therapies include PARP inhibitors, drugs targeting certain KRAS mutations, and TRK inhibitors.^5^ Unfortunately, PARP inhibitors, mainly used for treating tumors with DDR deficiencies, along with drugs targeting certain KRAS mutations and TRK inhibitors, are not effective for all patients.^6^ Immunotherapy, which has shown promise in various cancers,^7^ is currently effective only in a small subset of PDAC patients with deficient mismatch repair (dMMR) and/or high microsatellite instability (MSI-H).^8^ Overall, despite considerable efforts, the death rate among pancreatic cancer patients is still elevated, with a 5-year survival rate under 10%.^9^

The unique microenvironment of pancreatic cancer presents additional challenges in its treatment. Approximately 70% of pancreatic cancer patients exhibit a “dense” tumor microenvironment, characterized by a high concentration of dense connective tissue and extracellular matrix. This not only hampers effective drug delivery but may also weaken the effectiveness of chemotherapy and other treatment modalities. This microenvironmental feature renders pancreatic cancer a difficult-to-treat cancer type, and new drugs or new strategies that can overcome this limitation are urgently needed.

Aptamers, composed of short single-stranded DNA or RNA molecules, represent a compelling alternative. These molecules can be either selected or meticulously designed to exhibit potent affinity and specificity for particular target molecules, such as disease-associated proteins or cell surface receptors.^10^ Aptamers offer a broad spectrum of advantages, including ease of synthesis and customization, thermal stability, diminutive molecular size, and robust tissue penetration capabilities.^11^ These attributes render aptamers promising candidates for the delivery of therapeutic agents. Sgc8c is a biocompatible and non-toxic nucleic acid aptamer that specifically binds to PTK7, a protein overexpressed in pancreatic cancer tissues. Prior research highlights its effectiveness in targeting PTK7,^12^ making it a promising tool for selectively targeting pancreatic cancer cells and tissues.^13^ Recent years have seen significant advances in drug delivery methods, particularly through the strategic use of nucleic acid aptamers. This progress has led to the development of aptamer-drug conjugates (ApDC), innovative constructs that skillfully combine the targeting accuracy of aptamers with the therapeutic effectiveness of drug payloads. One such payload is Monomethyl Auristatin E (MMAE), a tubulin inhibitor that disrupts microtubule dynamics, leading to cell cycle arrest in the G2/M phase and subsequent apoptosis.^14^ The application of MMAE in constructing ApDCs (Aptamer-drug conjugates) has shown promise in targeted cancer therapy.^15^ However, it should be noted that nucleic acid aptamers have some drawbacks, such as the risk of renal clearance.^16^ and degradation by nucleases in vivo.^17^ This may culminate in premature drug exposure and unintended toxic consequences.^18^ Recent studies indicate that modifying nucleic acid aptamers or strategically conjugating them with microorganisms can significantly prolong their presence in the body and reduce their vulnerability to renal clearance.^19,20^ However, the biosafety of organisms needs to be considered.

VNP20009, characterized as an attenuated strain of *Salmonella*, exhibits a unique cell membrane-penetrating ability and the further capacity to navigate the “dense” stroma of the pancreas in addition to a distinct preference for anaerobic environments. Previous studies have demonstrated its ability to induce apoptosis and necrosis in tumor cells,^21^ while concurrently galvanizing the host’s immune system to mount a robust response, thereby effectively hindering tumor growth.^22^ However, Phase I clinical trials that utilized these bacteria as standalone treatments for tumors have not yielded substantial tumor regression, revealing a critical limitation: insufficient toxicity and targeting effect.^23^ Increasingly evidence suggests that bacterial therapy could be synergistically combined with other treatment modalities; meanwhile, the outstanding penetration noted above and biosafety make bacteria an intriguing candidate for nucleic acid-based drug delivery in the realm of solid tumor therapy.^24^

To overcome the significant challenges in pancreatic cancer treatment, we propose a novel synergistic therapy that harnesses the mutual benefits of bacteria and aptamer-drug conjugates (ApDC). Using a simple and direct “click chemistry” approach, we covalently attach ApDC to the surface of VNP20009. This innovative strategy leverages the mutual advantages of both components: ApDC enhances bacterial colonization within the tumor microenvironment (TME), while VNP significantly improves the stability of ApDC and assists in its effective penetration of the dense tumor stroma. This reciprocal relationship ensures that the therapeutic agent reaches the tumor more effectively and remains stable, leading to optimized drug delivery, enhanced therapeutic efficacy, and activation of a targeted immune response. This dual-functional approach offers a promising new direction for the treatment of pancreatic cancer.

## Results

### Construction and characterization of Sgc8c-MMAE-anchored VNP20009

To validate PTK7 as a target in pancreatic cancer, we conducted survival analysis, comparative expression studies using The Cancer Genome Atlas (TCGA) data, and immunohistochemical (IHC) staining. IHC showed high PTK7 expression in pancreatic cancer tissues across different stages (Supplementary Fig. 1a, b). Kaplan–Meier curves indicated that higher PTK7 expression correlates with poorer disease-free survival (Supplementary Fig. 1c, d). Comparative analysis revealed significantly higher PTK7 levels in cancerous versus non-tumor tissues (Supplementary Fig. 1e). These results confirm PTK7’s high expression in pancreatic cancer, supporting its potential for targeted therapy.

**Fig. 1:**
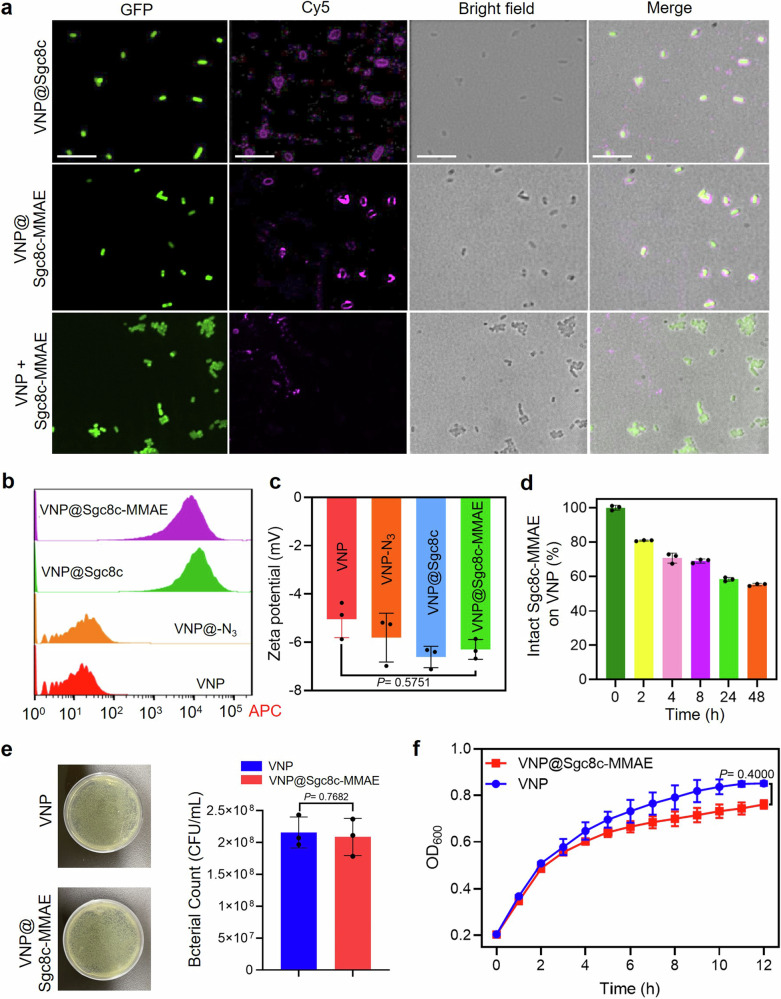
Construction of functionalized VNP@Sgc8c-MMAE. **a** Laser scanning confocal microscopy images of VNP@Sgc8c, VNP@Sgc8c-MMAE and VNP+Sgc8c-MMAE. Green and violet channels represent VNP_GFP_ and Cy5-labeled Sgc8c or Sgc8c-MMAE, respectively. Scale bar: 10 μm. **b** Flow cytometric analysis of fluorescence intensity in VNP20009 after 4 h of incubation with D-Azidoalanine and subsequently anchored with Cy5-labeled Sgc8c or Sgc8c-MMAE. **c** Zeta potentials of native VNP20009 and its surface-modified variants: VNP-N_3_, VNP@Sgc8c and VNP@Sgc8c-MMAE. One-way ANOVA analysis followed by Fisher’s LSD multiple comparison. **d** Degradation kinetics of Cy5-labeled Sgc8c-MMAE on VNP20009 exposed to PBS solution containing 20% fetal bovine serum at 37 °C. **e** Representative digital images of agar plates and corresponding colony counts of VNP and VNP@Sgc8c-MMAE after 12 h of culture in LB medium, followed by plating on LB agar. **f** Growth curves of native VNP20009 and VNP@Sgc8c-MMAE cultured in LB liquid medium. OD600 was recorded at 1 h intervals by microplate reader. Data are presented as mean ± s.d. (*n* = 3), with statistical significance assessed by two-tailed *t* test

Leveraging click chemistry’s efficiency for inert bond formation,^25^ we introduced Azido-D-alanine (–N_3_) into bacterial growth medium, leading to its integration into the bacterial cell wall and the presentation of azide groups (VNP–N_3_). To facilitate the conjugation process, we utilized Dibenzocyclooctyne (DBCO), a biocompatible linker commonly used in click chemistry for conjugating biomolecules. We then conjugated aptamer DBCO-Sgc8c to the drug monomethyl auristatin E (MMAE), following our established protocol,^26^ and subsequently confirmed the product DBCO-Sgc8c-MMAE by mass spectrometry (MS) (Supplementary Fig. 2). We observed that incubation of azide-modified bacteria with DBCO-modified Sgc8c-MMAE at 37 °C for 4 h facilitated a copper-free click reaction, resulting in the construction of functionalized (i.e., drug-loaded) bacteria (VNP@Sgc8c-MMAE). For visualization purposes, the Sgc8c-MMAE component was tagged with a Cy5 fluorescent probe, enabling the tracking of its co-localization with inherently GFP-expressing VNP20009 bacteria (VNP_GFP_) via laser scanning confocal microscopy (LSCM). Notably, both unmodified Sgc8c aptamer and its drug-conjugated variant, Sgc8c-MMAE, effectively bound to the VNP20009 bacterial surface (Fig. 1a). To exclude the probability of non-specific adsorption of nucleic acid to the VNP surface, we mixed VNP lacking N_3_ with Sgc8c-MMAE. LSCM results showed that there was no Cy5 fluorescence signal on the bacterial surface, confirming the conjugation via the “click” reaction. This binding was further corroborated by flow cytometry analysis, which demonstrated a marked increase in Cy5 fluorescence intensity on the functionalized bacteria (Fig. 1b). Additionally, the rise in Zeta-potential of functionalized bacteria indicated interaction with negatively charged ApDC, affirming the conjugation’s efficacy (Fig. 1c). Together, these results validate the efficacy and reliability of using click chemistry for conjugating ApDC to bacteria.

To quantify the number of aptamers anchored onto each bacterium, fluorescence spectrophotometric measurements were conducted on VNP20009 conjugated with Cy5-labeled Sgc8c-MMAE. As delineated in Supplementary Fig. 2a–c, we deduced the density of surface-bound Sgc8c-MMAE to be ~2.5 nmol of ApDC per 10^8^ CFU bacteria, equating to around 1.5 × 10^7^ Sgc8c-MMAE per VNP20009. Notably, this click chemistry-based nucleic acid-bacteria conjugation method displayed a yield that is 27-fold superior to the amide reaction previously documented in the literature.^19^

### The stability of ApDC is crucial for in vivo applications

To assess the stability of aptamer on bacteria, we monitored the fluorescence intensity of Cy5-labeled Sgc8c-MMAE on bacteria after incubation with 20% fetal bovine serum (FBS) in PBS at 37 °C for different time point (Supplementary Fig. 2d). Compared to our prior findings reporting that over 60% of free ApDC degraded within 12 h^18^ the bacterial surface-grafted ApDC exhibited enhanced stability, retaining over 60% of the aptamers intact after 48 h (Fig. 1d). We suspect that attaching ApDC onto the surface of bacteria could significantly enhance the steric hindrance, thereby reducing the nucleolytic degradation of ApDC.

To determine whether ApDC conjugation or preparation method influenced VNP20009 growth, both unmodified VNP20009 and VNP@Sgc8c-MMAE were cultured in 100% LB culture medium for 12 h. The bacterial counts of both groups were determined using the plate count method, and growth kinetics within 12 h were monitored via optical density measurements at 600 nm. The results suggested a negligible impact on bacterial growth. (Fig. 1e, f and Supplementary Fig. 2e). This corresponds to the understanding that the drug MMAE, as a mitosis inhibitor, primarily affects eukaryotic cells.^27^ Bacteria are prokaryotic organisms and undergo binary fission rather than mitosis, MMAE is unlikely to have the same direct inhibitory effect on bacteria.^28^

### VNP@ApDC function verification in vitro

Compared to other cancers, fewer actionable or “druggable” targets were found in pancreatic cancer. Studies have shown that PTK7 is overexpressed in several types of cancers, including pancreatic cancer,^12^ as demonstrated in our flow cytometry results (Supplementary Fig. 3a). To validate the specific binding of Sgc8c to pancreatic cancer, we utilized a random oligonucleotide library with the same number of bases as a control (Lib) and tested binding affinity with three different pancreatic cancer cell lines (Panc-1, Miapaca-2 and KPC1199). Cells were incubated with Cy5-labeled Lib, Sgc8c, or Sgc8c-MMAE on ice for 30 min, and flow cytometry results highlighted modest PTK7 expression in specific pancreatic cancer subsets (Supplementary Fig. 3a). Both native Sgc8c and its drug-conjugated version, Sgc8c-MMAE, exhibited prominent binding to pancreatic cancer cells (Supplementary Fig. 3b). Additionally, LSCM results indicated that Sgc8c effectively bound to all three pancreatic cancer cell lines and that its binding affinity was not compromised by MMAE conjugation (Supplementary Fig. 3c).

**Fig. 2:**
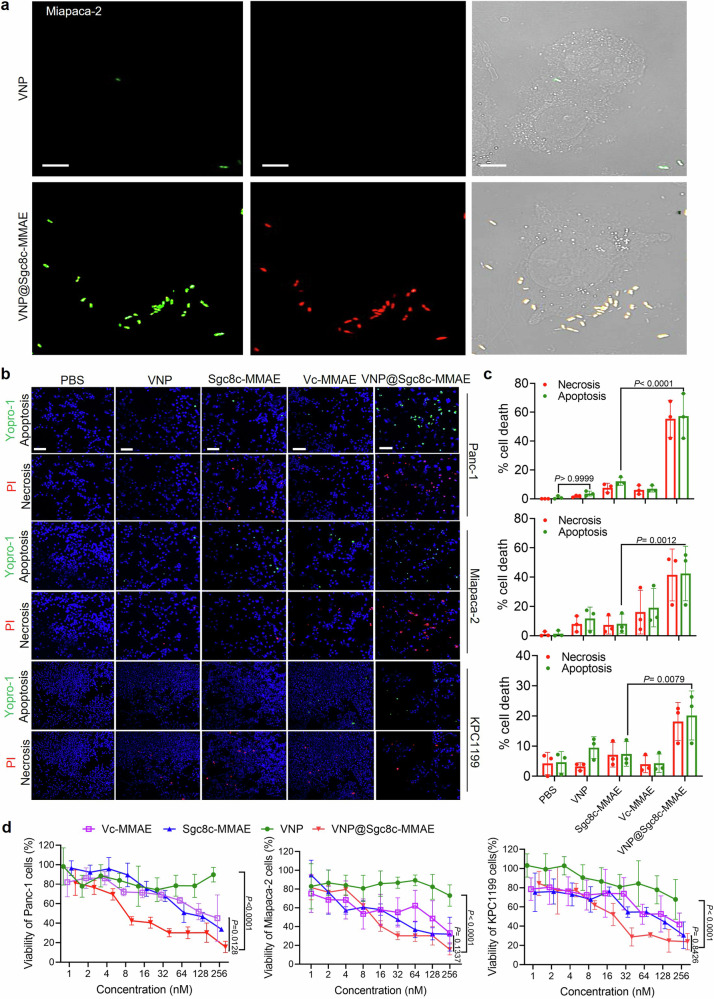
Targeting effect and cytotoxicity of functionalized VNP@Sgc8c-MMAE to pancreatic cancer cell lines. **a** LSCM images of Miapaca-2 pancreatic cancer cells incubated with 10^7^ CFU VNP20009 or VNP@Sgc8c-MMAE for 2 h at 37 °C. Cells were rinsed with DPBS three times before observation. Green and red channels respectively show VNP_GFP_ and Cy5-labeled Sgc8c-MMAE anchored on VNP_GFP_. Scale bar: 10 μm. **b** LSCM images and **c** quantification of cell death of pancreatic cancer cells stained with Hochest33342 (cell nuclei blue), Yopro-1 (apoptosis cells, green) and PI (necrosis cells, red) after incubation with VNP20009, Sgc8c-MMAE, Vc-MMAE or VNP@Sgc8c-MMAE at equal Vc-MMAE concentration (64 nM) for 48 h. Scale bar: 50 μm. **d** Viability of Panc-1, Miapaca-2, KPC1199 cells treated with VNP20009, Sgc8c-MMAE, Vc-MMAE or VNP@Sgc8c-MMAE at equal Vc-MMAE concentration (from 1 to 256 nM) for 72 h. VNP20009 concentration: 10^7^ CFU. Data are shown as mean ± s.d. of three independent experiments. Two-way ANOVA analysis followed by Fisher’s LSD multiple comparison

To further test whether VNP conjugation would influence the targeting effect of Sgc8c, LSCM was performed. Following a 2-h coincubation with VNP@Sgc8c-MMAE and naive VNP20009, LSCM visualization revealed an increased bacterial association with the Miapaca-2 cells, underscoring the contributory role of Sgc8c-MMAE (Fig. 2a and Supplementary Fig. 4).

The synergistic cytotoxicity of VNP@Sgc8c-MMAE on pancreatic cancer cells was studied with the Yopro-PI assay (Fig. 2b). Results indicated only negligible cytotoxicity from standalone VNP20009. In contrast, VNP@Sgc8c-MMAE, benefiting from both targeting toxicity of ApDC and its intrinsic antitumor potency, triggered 20–60% cell death (necrosis and apoptosis) at the corresponding bacterial concentrations (Fig. 2b, c). The differences in cell death among the three pancreatic cancer cell lines may be attributed to PTK7 protein levels, with lower PTK7 on KPC1199 cells leading to reduced uptake of VNP@Sgc8c-MMAE and consequently lower cytotoxic effects, which is in line with the results in Supplementary Fig. 3d–f and Supplementary Fig. 4c.

Simultaneously, a collaborative synergy was observed between bacteria and drug. With the concentration of MMAE maintained at 64 nM, the standalone drug, Sgc8c-MMAE, triggered roughly 10% cell apoptosis and necrosis. On the other hand, the VNP@Sgc8c-MMAE group caused about 2–8 times more cell death compared to the Sgc8c-MMAE group among the three cell lines. Cytotoxicity of VNP@Sgc8c-MMAE was further explored with the CCK-8 assay, which evaluated the influence of different drug concentrations on the viability of pancreatic cancer cells. Notably, the VNP@Sgc8c-MMAE group demonstrated a markedly heightened cytotoxicity, which was dose-dependent, compared to the other groups (Fig. 2d).

The efficiency of bacterial penetration was also confirmed in a Miapaca-2 tumor xenograft model, followed by intravenous injection of PBS or 1 × 10^7^ CFU of GFP-expressing VNP20009 or VNP@ApDC. Tumor tissues were collected and sections were prepared for fluorescence microscopy study. ApDC increased the colonization of VNP20009 at the tumor site, resulting in a corresponding increase in the quantity of VNP@Sgc8c-MMAE inside the tissue in comparison to other control groups (Supplementary Fig. 5).

In summary, VNP@ApDC maintained its aptamer-targeting effect, drug cytotoxicity, and bacterial penetration to ensure its in vivo application.

### ApDC-assisted bacteria efficiently colonize in vivo

**Fig. 3:**
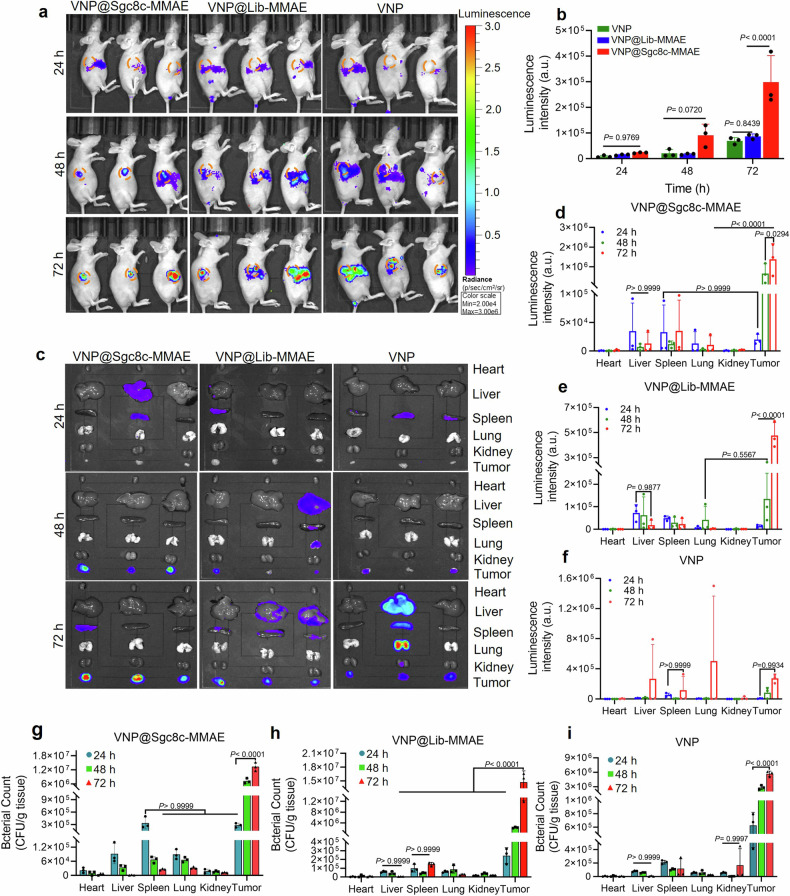
Targeting enrichment of VNP@Sgc8c-MMAE at tumor site. **a** IVIS imaging of Miapaca-2 tumor-bearing mice at 24, 48 and 72 h after i.v. injection with 10^7^ CFUs of native Lux-engineered VNP, VNP@Lib-MMAE or VNP@Sgc8c-MMAE (*n* = 3 mice per group). **b** Average intensity of luminescent signals from circled tumor region (orange) at 24, 48 and 72 h calculated by IVIS Lumina II system. **c** Ex vivo IVIS Lumina imaging and **d**–**f** average luminescence intensity in tumor and major organs of Miapaca-2 tumor-bearing mice from 24 h to 72 h after i.v. injection with Lux-engineered VNP, VNP@Lib-MMAE or VNP@Sgc8c-MMAE. Bacterial counts of (**g**) VNP@Sgc8c-MMAE, **h** VNP@Lib-MMAE and **i** VNP in tumor and major tissues from 24 h to 72 h after first treatment. Data are presented as mean ± s.d. (*n* = 3 mice per group, two-way ANOVA analysis followed by Fisher’s LSD multiple comparison

To verify application potential, bacteria were tracked in vivo. To accomplish this, VNP@Sgc8c-MMAE, engineered with the LuxCDABE cluster, was intravenously injected to Miapaca-2 tumor-bearing mice, alongside controls Lux-engineered VNP20009 and VNP@Lib-MMAE. In vivo imaging results showed increasing luminescence at tumor locations from 24 to 72 h, and VNP@Sgc8c-MMAE displayed a threefold higher signal than controls at 72 h (Fig. 3a, b). While controls indicated accumulation in multiple organs, the tumor-specific localization of VNP@Sgc8c-MMAE suggests the effect of specific aptamer-targeting to pancreatic cancer. Comparable outcomes were also observed in the ex vivo results (Supplementary Fig. 6a–d).

To elucidate the biodistribution of bacteria, we conducted ex vivo imaging of organs from an additional 9 mice. Heart, kidney, liver, lungs, spleen, and tumor tissues were collected from mice and homogenized, and the homogenate was coated on LB agar for bacterial proliferation, and the number of bacteria was counted. At 24 h, bacteria had mainly colonized in the liver and spleen, but eliminated within 48 h (Fig. 3c–i). Given the preference of bacteria for the hypoxic TME and the specific binding of Sgc8c to pancreatic cancer cells, nearly 90% VNP@Sgc8c-MMAE colonized the tumor at 72 h. Meanwhile, unmodified VNP or VNP@Lib-MMAE showed some presence in the liver, kidneys, and spleen, but the signal intensity was lower than that of the VNP@Sgc8c-MMAE group. This indicates that the conjugation of Sgc8c-MMAE to VNP does not compromise the bacteria’s innate targeting ability. Instead, it enhances their specific localization at PTK7-overexpressing tumor tissues. Additionally, the luminescence signal of VNP@Sgc8c-MMAE at tumor sites was 4–5 times brighter than that of the VNP group at 72 h (Fig. 3d–f), according to the in vivo results.

To further investigate the in vivo stability of ApDC, we used luminescence and fluorescence imaging in immune-competent KPC1199 tumor-bearing C57 mice to colocalize ApDC with VNP (Supplementary Fig. 6e). The results indicated that the majority of Cy5-labeled ApDC signals remained in the tumor region after 72 h, demonstrating significant stability and accumulation at the tumor site (Supplementary Fig. 6f, i). The VNP carrier notably enhanced ApDC stability, maintaining its presence in the tumor area longer compared to unmodified nucleic acid drugs, which typically have a short half-life in vivo.^29^

Furthermore, Supplementary Fig. 6g, h shows that VNP@Sgc8c-MMAE exhibited higher stability and accumulation than other groups. The ApDC facilitated bacterial colonization at the tumor site, while the bacteria enhanced ApDC accumulation in the tumor, addressing the suitability and efficacy of the VNP@Sgc8c-MMAE combination.

### Bacteria-assisted deep penetration of ApDC in tumor

**Fig. 4:**
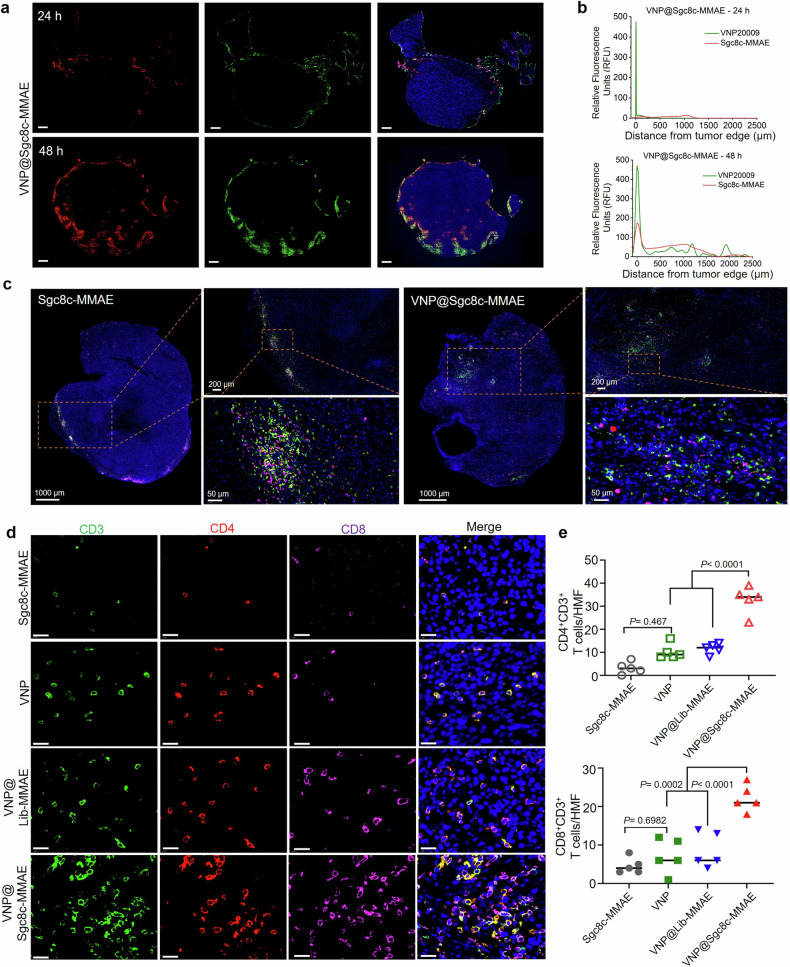
Multifaceted analysis of immune response and drug delivery in pancreatic cancer tissues. **a** Confocal images of tumor tissue sections collected from KPC1199 subcutaneous tumor-bearing mouse at 24 h and 48 h post-intravenous injection of 10^7^ CFU VNP@Sgc8c-MMAE-Cy5. VNP_GFP_ is depicted in green, nuclei stained with DAPI are shown in blue, and Cy5-labeled Sgc8c-MMAE appear in red. Scale bar: 500 μm. **b** Quantification of payload penetration. The green represents VNP_GFP_, and the red represents Cy5-labeled Sgc8c-MMAE. **c** Representative confocal images depicting immune cell infiltration in KPC1199 subcutaneous pancreatic tumor tissues after intravenous administration of Sgc8c-MMAE or VNP@Sgc8c-MMAE. The orange box marks the area selected for obtaining the high-magnification field (HMF) image. **d** Representative confocal images of immune cells in tumor tissue of a pancreatic cancer in-situ carcinoma model. Scale bar: 20 μm. Blue indicates cell nuclei stained with DAPI, green represents CD3 immune cells, purple highlights CD8^+^ T cells, and red signifies CD4^+^ T cells. **e** Quantitative analysis of CD4^+^CD3^+^ T cells and CD8^+^ CD3^+^ T cells within the tumor based on cell counts in each high-magnification field within both tumor and adjacent non-tumor regions. Analysis conducted on five representative fields per section using ImageJ software. Results are presented as mean ± standard error of the mean (one-way ANOVA analysis, followed by Fisher’s LSD multiple comparison)

Pancreatic cancer possesses a dense, intricate microenvironment.^30^ that obstructs drug and immune cell penetration, thereby limiting the efficacy of chemotherapy and immunotherapy.^31^ Prior research suggests that VNP20009 can navigate through the defenses of pancreatic tumors to penetrate deeply,^32^ which has been proved in an Miapaca-2-bearing CDX model. The penetrative effect of VNP@Sgc8c-MMAE was further confirmed in KPC1199 subcutaneous mouse model (Fig. 4 and Supplementary Fig. 7), Patient-Derived Xenograft (PDX) mouse model (Supplementary Fig. 8) and in-situ carcinoma model (Supplementary Fig. 9), each administered with Sgc8c-MMAE or VNP@Sgc8c-MMAE. Tumor tissues were extracted 24 or 48 h later, and the co-localization effect of VNP_GFP_ and Cy5-Sgc8c-MMAE was observed. Figure 4a illustrates how bacteria (green) ferry the drug (red) deeper into the tumor over time, while the individual drug, Sgc8c-MMAE, lingers at the tumor’s periphery 48 h post-injection. The quantification of fluorescence intensity distribution revealed distinct patterns of drug and bacterial localization within tumor spheroids.

For the VNP@Sgc8c-MMAE group, Fig. 4b shows that a subset of bacteria and drugs colocalized at a significant distance, ~1000 μm from the tumor’s edge. In contrast, the distribution of the drug alone, Sgc8c-MMAE, was predominantly confined to a region within 30 μm of the tumor’s edge (Supplementary Fig. 7). These observations collectively suggest a role for bacteria as a vector for deep tumor penetration by therapeutic agents. This may be attributed to the bacteria’s innate flagellar motility, which allows navigation through tissues unaffected by fluid dynamics or hydrodynamic forces.^33^

### Tumor microenvironment improvement induced by VNP@ApDC penetration

With the colonization of bacteria and the penetration of ApDC, drug release was confirmed by LSCM. In addition, the detachment of ApDC from bacteria was evidenced by the distribution of Cy5 fluorescence (Supplementary Fig. 10). The ability of bacteria to degrade proteins and polysaccharides in the tumor extracellular matrix^34,35^ was reported previously; however, with the proliferation of bacteria and release of the drug, we propose an improvement in the tumor microenvironment. To verify this hypothesis, we first assessed tissue density using α-SMA and Masson staining, commonly utilized to identify pathologic fibroblasts associated with the dense microenvironment of pancreatic cancer. Supplementary Figs. 8 and 9 demonstrate decreased staining in the VNP@Sgc8c-MMAE group. We suspect that a relatively loose TME may be created by VNP@ApDC, which may facilitate the infiltration of T cells. To confirm this, immunofluorescence assays were performed with anti-CD4, anti-CD8, and anti-CD3 antibodies on tumor tissue sections obtained from different treatments in a pancreatic cancer subcutaneous tumor mouse model. As depicted in Fig. 4c, we observed an enrichment of T cells in tumor center after injection of VNP@Sgc8c-MMAE. Tumor tissues of the KPC1199 in-situ carcinoma model 14 days after administration were also collected for IHC analysis. Results revealed that the VNP@Sgc8c-MMAE group had 3–4 times the number of CD4^+^ T cells and CD8^+^ T cells compared to the control group (Fig. 4d, e) in agreement with the in vivo targeting results.

At the same time, scRNA-seq analysis demonstrated a significant increase in cytotoxic CD8^+^ T cells and helper CD4^+^ T cells in the VNP@ApDC group, indicating an enhanced immune response. Additionally, there was a marked reduction in myofibroblastic CAFs (myoCAFs), which typically impede immune cell infiltration by remodeling the extracellular matrix and promoting fibrosis. This reduction likely facilitated the increased infiltration of immune cells. These findings suggest that VNP@Sgc8c-MMAE boosts antitumor immunity not only by increasing cytotoxic and helper T cell populations but also by potentially modifying the tumor stroma to alleviate barriers to immune cell infiltration, highlighting its potential as a powerful immunotherapeutic strategy (Supplementary Fig. 11).

These findings, in alignment with the results of bacterial colonization and ApDC penetration confirm the synergistic effect of the bacteria-ApDC complex in these models.

### In vivo synergistic therapy efficacy of VNP@ApDC

**Fig. 5:**
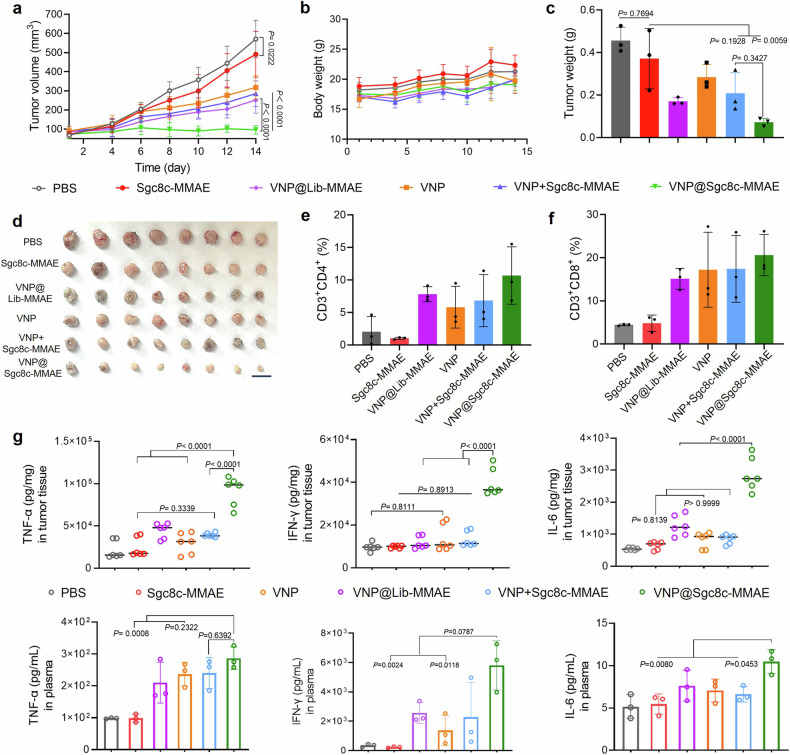
In vivo therapeutic efficacy in KPC1199 subcutaneous pancreatic tumor model. **a** The change of tumor volume as a function of time after different treatments (*n* = 8). **b** Fluctuation of body weight after treatment (*n* = 8). **c** Tumor weight of different treatments on the day of sacrifice. **d** Representative digital photo of tumor tissues harvested from C57 mice on the day of sacrifice. Flow cytometric analysis of the population of **e** CD4^+^ and **f** CD8^+^ T cells gated on CD3^+^ T cells within tumor tissues (*n* = 3). **g** Quantification of TNF-α, IFN-γ and IL-6 in tumor tissue lysate and plasma of KPC1199-bearing C57 mice at the end of treatment. Data are shown as mean ± s.d. One-way ANOVA analysis followed by Fisher’s LSD multiple comparison

To assess the efficacy of in vivo synergistic therapy in bacteria-assisted drug delivery, we first established a subcutaneous pancreatic tumor model with KPC1199 cells. Mice were divided into six groups (PBS, Sgc8c-MMAE, VNP, VNP+Sgc8c-MMAE, VNP@Lib-MMAE and VNP@Sgc8-MMAE, *n* = 8), and each group received intravenous injections of bacterial concentrations (10^7^ CFU) and drug dosages (0.25 nmol). Results revealed that VNP-containing formulations consistently impeded tumor progression (Fig. 5a–d). Particularly, the VNP@Sgc8c-MMAE group displayed superior antitumor activity compared to controls. Body weight tracking indicated negligible affection on mouse health with different treatment at the given dosages (Fig. 5b).

To further explore the immune reaction triggered by VNP@ApDC, T-cell activation was analyzed. A single-cell suspension was first prepared from tumor tissues post-treatment and studied with flow cytometry. With VNP@Sgc8c-MMAE treatment, results show a significant increase of CD3^+^ CD4^+^ T cells in the VNP group and the combined VNP@ApDC group. In contrast, no notable T-cell infiltration was found in either the PBS or Sgc8c-MMAE groups (Fig. 5e, f and Supplementary Fig. 12). Cytokines are always secreted upon T cell activation, so to further assess the synergistic therapy of VNP@ApDC, ELISA was performed to test the level of interferon-γ (IFN-γ) and tumor necrosis factor-α (TNF-α). Results revealed that the level of both was elevated in plasma and tumor tissues (Fig. 5g), suggesting that VNP@Sgc8c-MMAE treatment had activated a heightened antitumor immune response compared to bacteria and ApDC.

Additionally, hematoxylin and eosin (H&E) staining was carried out to monitor tissue necrosis (Supplementary Fig. 13). Results revealed significant necrosis in tumor tissues after treatment with the VNP@ApDC. Ki67 staining underscored the marked inhibitory effect of VNP@ApDC on tumor cell proliferation. TUNEL assay highlighted that the VNP@Sgc8c-MMAE group induced the most pronounced cancer cell death (Supplementary Fig. 14).

**Fig. 6:**
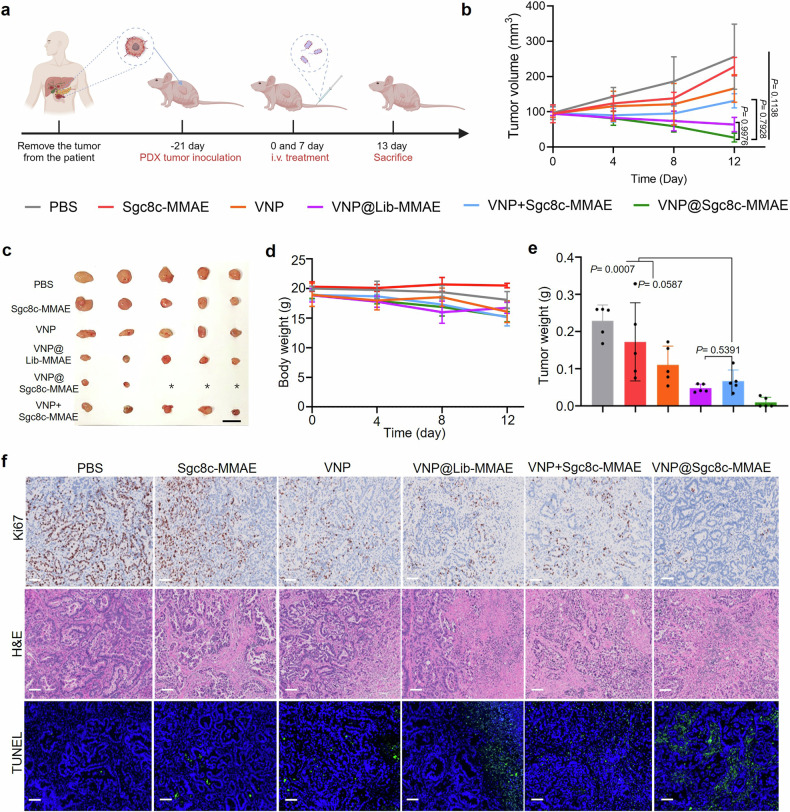
In vivo therapeutic efficacy in a PDX model. **a** PDX model construction and treatment flowchart. Image created with Biorender.com, with permission. **b** Tumor volume variation over time following various treatments (*n* = 5). Results are presented as the mean ± s.d. from five separate trials. Utilizing a one-way ANOVA followed by Fisher’s LSD multiple comparison, significance values were determined as follows: *P* = 0.1138 for PBS vs. VNP@Sgc8c-MMAE and *P* = 0.7928 for VNP@Sgc8c-MMAE vs. VNP+Sgc8c-MMAE. **c** Representative images of tumor samples from mice taken post-treatment. Scale bar: 1 cm. **d** Body weight changes post-treatment (*n* = 5). **e** Tumor weights on the termination day across different treatments. **f** H&E, Ki67 and TUNEL (green) analysis of tumor samples. Scale bar: 100 μm

To further clarify the antitumor effect of bacteria-facilitated ApDC, a PDX model was utilized. Once tumors grew to a volume of around 100 mm^3^, mice were subjected to different therapeutic regimens, as depicted in Fig. 6a. Even in nude mice with a diminished immune response, results show a marked therapeutic advantage favoring the VNP@Sgc8c-MMAE group over the isolated Sgc8c-MMAE group (Fig. 6b–e and Supplementary Fig. 15). This differential outcome may hinge on the propensity of bacteria for the hypoxic TME. Notably, pancreatic tumors are often characterized by compromised blood perfusion, posing challenges for effective drug delivery to the tumor locus.^36,37^ In this context, bacteria can ferry a larger cache of ApDC into the tumor’s oxygen-deprived zones, thereby bolstering drug density within the tumor milieu. IHC assessments (Fig. 6f) vividly demonstrate that the VNP@ApDC group, relative to other groups, induced heightened cell death and decisive inhibition of tumor cell proliferation.

It is noteworthy that regressive tumor effect in the PDX model was more pronounced than that in the C57 subcutaneous tumor model constructed using KPC1199 cells. A possible explanation is the rapid growth of murine-derived KPC1199 cells, while the PDX model exhibits slower tumor progression.

**Fig. 7:**
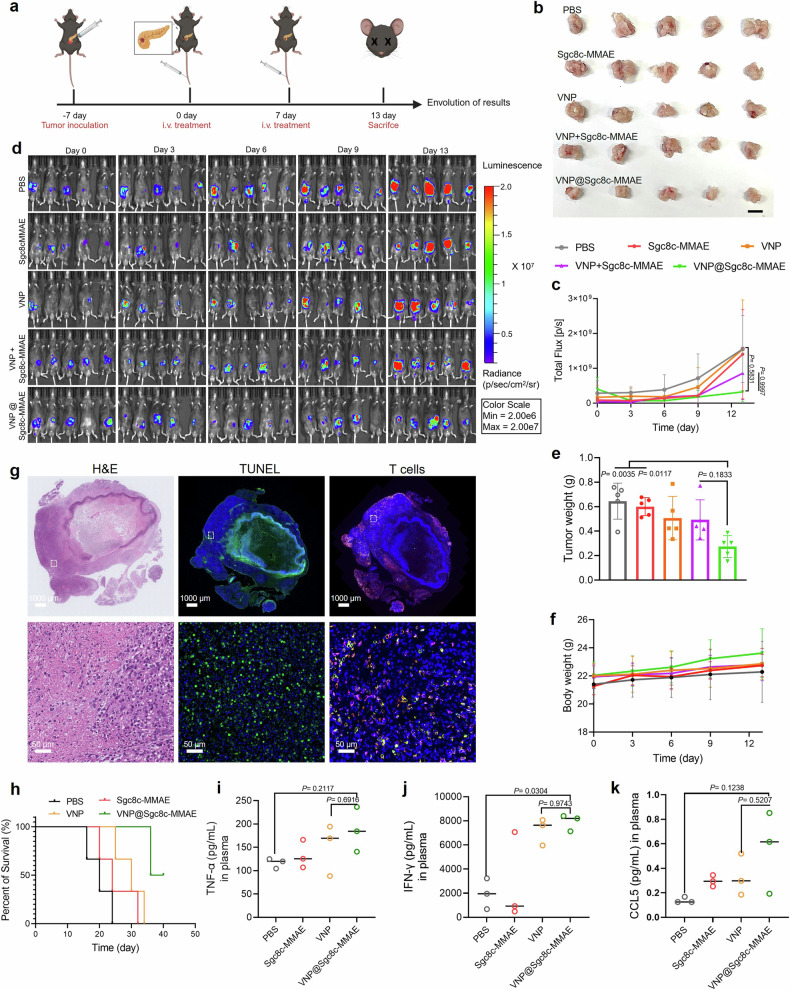
In vivo therapeutic efficacy in an in-situ carcinoma model. **a** Diagram illustrating the setup and treatment protocol of the in-situ carcinoma model of pancreatic cancer. Image created with Biorender.com, with permission. **b** Characteristic images of tumor tissues extracted from mice after treatment interventions. Scale bar: 1 cm. **c** Alterations in overall tumor flux as a function of time across distinct therapeutic strategies (*n* = 5). Data are depicted as mean ± s.d. from five independent experiments. **d** Luminescence intensity variations in pancreatic tumors of mice monitored using a live imaging system. **e** Recorded tumor masses at the study’s conclusion for each treatment group. **f** Body weight fluctuations observed post-treatment interventions (*n* = 5). **g** Tumor sections from the VNP@Sgc8c-MMAE group were analyzed using H&E, TUNEL (green) stains, and T cell stains. Cell nuclei were stained with DAPI (blue), CD3^+^ immune cells were stained with GFP-labeled anti-CD3 antibody (Green), and CD4^+^ immune cells were stained with APC-labeled anti-CD4 antibody (Red). CD8^+^ immune cells were stained with PI-labeled anti-CD8 antibody (Purple). Scale bar: 1000 μm (top) and 50 μm (bottom). The white box in the top image represents the area shown in the bottom image. **h** Survival curves for mice treated with PBS, Sgc8c-MMAE, VNP, and VNP@Sgc8c-MMAE. Cytokine levels in plasma of treated mice: **i** TNF-α, **j** IFN-γ, **k** CCL5. Statistical analysis was carried out using one-way ANOVA succeeded by Fisher’s LSD post-hoc test

To more closely emulate clinical scenarios of tumor development, progression, and therapeutic intervention, we initiated an in-situ carcinoma model of pancreatic cancer.^38^ The experiment entailed the implantation of the luciferase-expressing KPC1199 cell line into the pancreatic tissue of male C57 mice, as delineated in Fig. 7a. Tumor growth was analyzed by monitoring the bioluminescence signal. Results showed that the VNP@Sgc8c-MMAE group, which utilized bacteria-mediated drug delivery, demonstrated significant antitumor effects, as indicated by the reduced tumor size as shown in Fig. 7b–d.

Tumors in this group were substantially smaller, weighing only 30 to 35% of those in the Sgc8c-MMAE group and about half the size of those in other control groups (Fig. 7e). Notably, weight profiles and survival curve of the treated mice suggested no adverse effects (Fig. 7f–h). Additionally, the IFN-γ and TNF-α profiles suggest that VNP@Sgc8c-MMAE not only triggers a strong innate immune response but also enhances adaptive immunity through the induction of Th1-type cytokines (Fig. 7i, j). Specifically, as shown in Fig. 7k, higher CCL5 levels in the VNP@Sgc8c-MMAE group indicate its role in recruiting T cells to the tumor site, thereby enhancing the immune response and creating a more favorable environment for immunotherapy.

**Fig. 8:**
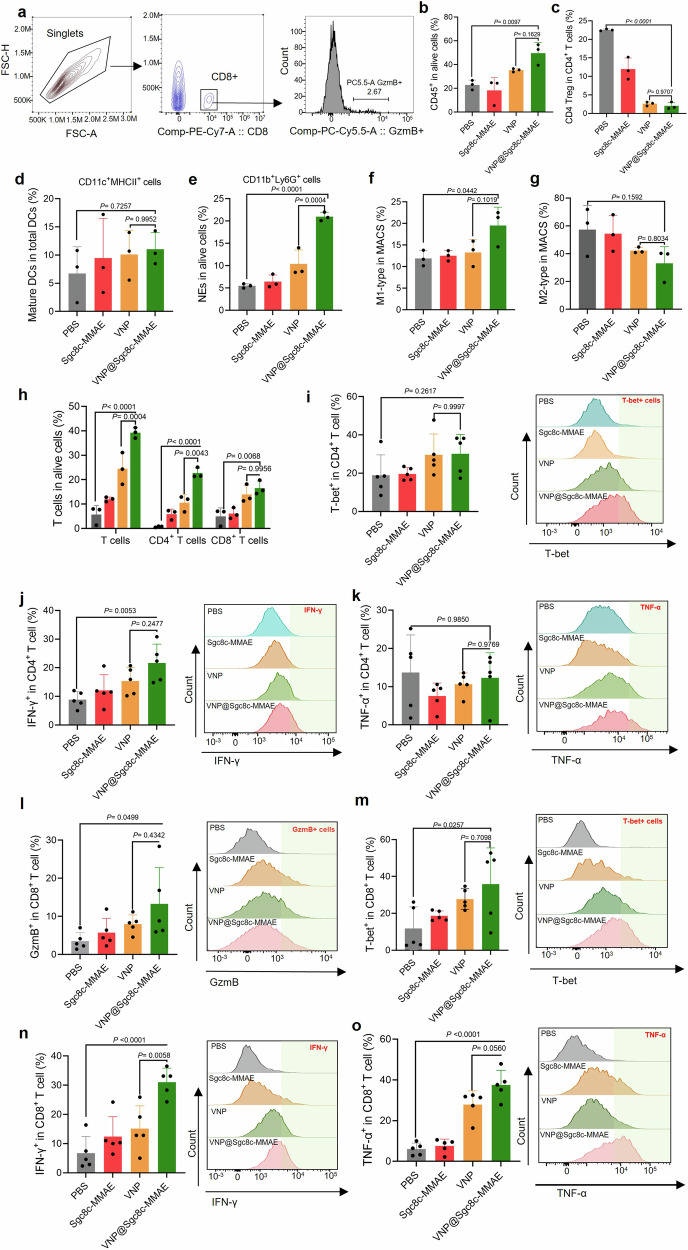
VNP@Sgc8c-MMAE activates antitumor immunity. **a** Gating strategy for flow cytometry analysis of immune cells, highlighting singlets, CD8^+^ cells, and GzmB^+^ cells. **b** Percentage of CD45^+^ immune cells in tumors after different treatments. **c** Percentage of CD4^+^ T regulatory (Treg) cells (CD25^+^CD127^-^) in the CD4^+^ T cell population in tumors after different treatments. The VNP@Sgc8c-MMAE group shows a significant decrease in CD4^+^ Treg cells compared to the PBS control. Analysis of various immune cell populations, including **d** mature dendritic cells (CD11c^+^MHCII^+^), **e** neutrophils (CD11b^+^Ly6G^+^), **f** M1-type macrophages, and **g** M2-type macrophages, with VNP@Sgc8c-MMAE treatment showing significant increases in M1-type macrophages and decreases in M2-type macrophages. **h** Proportion of total T cells, CD4^+^ T cells, and CD8^+^ T cells in live cell populations, demonstrating significant increases in T cell infiltration in the VNP@Sgc8c-MMAE group. **i** T-bet expression in CD4^+^ T cells, indicating Th1 differentiation. **J**, **K** IFN-γ and TNF-α production in CD4^+^ T cells, with significant upregulation in the VNP@Sgc8c-MMAE group. **l**, **m** Granzyme B (GzmB) and T-bet expression in CD8^+^ T cells, showing enhanced cytotoxic activity and Th1 differentiation. **n**, **o** IFN-γ and TNF-α production in CD8^+^ T cells, indicating enhanced immune responses in the VNP@Sgc8c-MMAE group. Data are presented as the mean ± s.d. (*n* = 3–5)

To assess the immune response elicited by VNP@Sgc8c-MMAE, tumor tissues from treated mice were processed into single-cell suspensions and analyzed via flow cytometry. The analysis included CD45^+^ immune cells, CD4^+^ and CD8^+^ T cells, dendritic cells, neutrophils, and M1 and M2 macrophages. Functional markers such as T-bet, IFN-γ, TNF-α, and granzyme B (GzmB) were also measured. The gating strategy for flow cytometry analysis was established to ensure accurate identification and quantification of immune cell populations (Fig. 8a).

Results demonstrated a significant increase in the infiltration of CD45^+^ immune cells in tumor tissues treated with VNP@Sgc8c-MMAE compared to control groups (Fig. 8b), indicating a robust immune response facilitated by the bacterial carrier. Analysis of CD4^+^ T cells showed enhanced infiltration in the VNP@Sgc8c-MMAE treated group, with a marked decrease in CD4^+^ Tregs, suggesting a reduction in immunosuppressive cell populations within the tumor microenvironment (Fig. 8c). There was a significant increase in mature dendritic cells (CD11c^+^MHCII^+^) in tumors treated with VNP@Sgc8c-MMAE, indicating enhanced antigen presentation capacity (Fig. 8d). Neutrophil (CD11b^+^Ly6G^+^) presence was also significantly elevated in the VNP@Sgc8c-MMAE group (Fig. 8e). The ratio of M1-type to M2-type macrophages increased in the VNP@Sgc8c-MMAE group, with M1 macrophages more prevalent and M2 macrophages less frequent, indicating a shift towards a pro-inflammatory macrophage phenotype (Fig. 8f, g).

The analysis of total T cells, including CD4^+^ and CD8^+^ T cells, showed a significant increase in the VNP@Sgc8c-MMAE treatment group (Fig. 8h). CD8^+^ T cells exhibited higher expression of GzmB, T-bet, IFN-γ, and TNF-α (Fig. 8l–o), indicating enhanced cytotoxic activity and a stronger Th1 immune response. These findings suggest that VNP@Sgc8c-MMAE effectively enhances both immune cell infiltration and functional antitumor immunity, contributing to boosting immune activation for antitumor function through several mechanisms: enhancing antigen presentation by APCs, increasing the production of antitumor cytokines and chemokines, and promoting a pro-inflammatory immune environment.

## Discussion

**Fig. 9:**
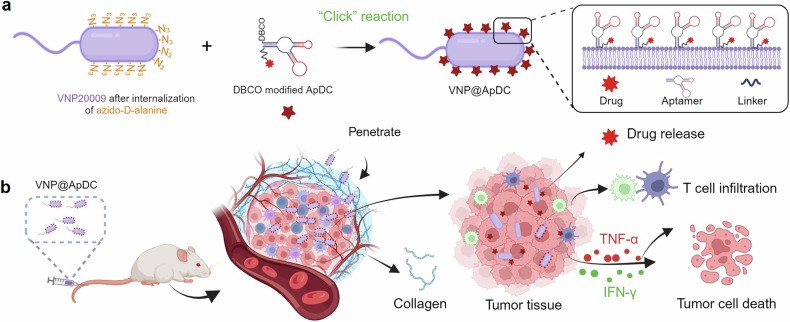
Schematic illustrating the construction of functionalized bacteria and the mechanism against pancreatic tumors. **a** Preparation of ApDC-anchored VNP20009 via a simple one-step click chemistry process. **b** Drug-loaded bacteria penetrate the stromal barriers of pancreatic neoplasms after intravenous (i.v.) administration, effectuating deep-tissue drug release. Concurrently, they secrete cytokines, triggering both apoptosis and necrosis in malignant cells, while amplifying the recruitment of immune cells to the tumor site. Image created with Biorender.com, with permission

Here, we developed a synergistic therapy by covalently attaching ApDC to the surface of VNP20009, generating drug-loaded bacteria. This approach capitalizes on the advantages of both bacteria and ApDC, allowing bacteria to act as delivery vehicles that target the tumor microenvironment (TME). The bacterial affinity for hypoxic conditions enables ApDC to penetrate deep into the tumor, enhancing drug delivery and therapeutic efficacy. Additionally, bacterial proliferation at the tumor site stimulates an immune response, offering a combined therapy against pancreatic cancer (Fig. 9).

The research presented in this study introduces an innovative combination of therapy modalities for pancreatic cancer, skillfully intertwining chemotherapy and immunotherapy. The application of ApDC (aptamer-Drug-Conjugates) labeled on bacteria represents a novel approach, capitalizing on the inherent tumor-homing properties of bacteria. This strategy not only enhances the delivery of therapeutic agents to the tumor site but also presents a potential solution to the limitations faced by conventional drug delivery systems in pancreatic cancer treatment, particularly those involved in overcoming the barriers posed by the TME.

This project selected VNP20009 as the carrier, MMAE as the drug, and Sgc8c as the targeting agent. VNP20009 was chosen for its safety and tumor-targeting capabilities, demonstrated in Phase I clinical trials, and its preferential accumulation in hypoxic tumor environments. MMAE was chosen for its efficacy as a well-validated cytotoxic agent used in antibody-drug conjugates (ADCs). Sgc8c was chosen for its ability to target PTK7, which is overexpressed in pancreatic cancer, and for being easier to modify than antibodies, thereby enhancing treatment precision and efficacy.

The role of bacteria in enhancing the stability and delivery of ApDC is a pivotal aspect of this study. In pancreatic cancer, dense stromal tissue often poses a significant challenge to effective drug delivery. Here, the natural inclination of bacteria towards hypoxic environments, which are characteristic of tumor sites, is leveraged to ensure the direct transport of ApDC to the tumor. This results in a more focused and potent therapeutic impact, crucial for a disease where traditional delivery methods frequently fall short.

A critical innovation in this study is the synergy created between the aptamer component and bacterial colonization. Surface markers for PDAC cells are selected using databases like TCGA and validated with tissue microarrays, while high-affinity aptamers are identified through SELEX technology. The aptamer’s specific affinity for cancer cells not only aids in targeting the tumor with precision but also enhances bacterial colonization at the tumor site. This dual-targeting mechanism is likely to substantially improve the treatment’s efficacy by ensuring a higher concentration of therapeutic agents directly where they are most needed.

The mechanism of tumor cell destruction and subsequent immune activation outlined in this study is particularly noteworthy. The therapy employs a dual approach: directly killing tumor cells via the release of drugs from VNP@ApDC and recruitment of T cells by the proliferation of bacteria. This not only targets the tumor cells but also initiates a transformative process in the TME. The subsequent recruitment of T cells, driven by the disruption of tumor cells and bacterial proliferation, fosters a more penetrative and active immune response within the deeper layers of the tumor tissue. Importantly, this study had considered the role of the immunosuppressive environment in pancreatic ductal adenocarcinoma (PDAC) tumors. The immunosuppressive environment of PDAC tumors may have contributed to the survival and proliferation of *Salmonella* within the tumor. This immunosuppressive state was lacking in normal tissues, allowing *Salmonella* to preferentially accumulate within the tumor microenvironment.

An essential aspect of this therapy is its potential to modify the TME. By breaking down tumor cells and encouraging bacterial proliferation, the treatment alters the microenvironment to favor immune cell infiltration and activity. This alteration is particularly crucial in the context of pancreatic cancer wherein the TME typically acts as a protective haven for the tumor and poses a significant obstacle to conventional treatments.

Despite the study’s primary focus on the therapeutic effects on primary tumors, one of the key challenges of PDAC is that the tumor often metastasizes before the primary tumor is detected. Based on the targeting properties of VNP@Sgc8c-MMAE and its effectiveness in primary tumors, we speculate that it may also exhibit similar targeting accumulation and antitumor effects in metastatic tumors. Future studies will be necessary to further validate the efficacy of VNP@Sgc8c-MMAE in metastatic tumor models.

While this study proposes a highly promising therapeutic strategy, further research is essential to fully understand its efficacy and safety. Future clinical trials will be crucial in determining the practical applicability of this approach and assessing its potential to enhance the survival rates and quality of life for pancreatic cancer patients.

VNP20009 was administered intravenously in Phase I clinical trials. The safety of bacterial injections is supported by clinical trials showing tolerability. However, alternative administration routes such as oral administration and fecal transplantation should be explored to minimize systemic immune activation and offer a more targeted approach. The impact of fecal transplantation on the microbiome must be carefully evaluated to ensure therapeutic benefits outweigh any disruptions to the natural microbial balance. Such studies are crucial for understanding the comprehensive safety profile and efficacy of VNP@Sgc8c-MMAE in a clinical setting. Future research will focus on optimizing bacterial strains, refining aptamer design, exploring suitable administration routes, and exploring combination therapies for better outcomes.

In conclusion, the study sets forth a highly promising and innovative therapeutic strategy that holds the potential to significantly impact pancreatic cancer treatment. By effectively combining different therapeutic modalities and overcoming the tumor microenvironment, this approach could address and potentially overcome some of the most significant challenges in the current landscape of pancreatic cancer treatment.

## Materials and methods

The related biological and chemical products were summarized as follows:

3-Azido-D-alanine hydrochloride (Baseclick GmbH, Inc.), Maleimidocaproyl-Val-Cit-PABC-monomethyl auristatin E (Monmouth Junction, Inc.), Tris(2-carboxyethyl) phosphine hydrochloride (TCEP, Sigma Aldrich), Anti-h/rPTK7/CCK4 Alexa Fluor® 488-conjugated Antibody (R&D Systems Co. Ltd.), Anti-rabbit IgG, F(ab’)2 Alexa Fluor® 488 Conjugate (Cell Signaling Technology, Inc.), Lysogeny broth (LB, BBI Life Science), Dulbecco’s Modified Eagle’s Medium (DMEM, Gibco), Fetal bovine serum (FBS, Gibco). Panc-1 cells (CRL-1469) and, Miapaca-2 cells (CRL-1420) were both from ATCC. The KPC1199 cell line was isolated from a spontaneous PDAC mouse model (LSL-KrasG12D, Pdxcre, LSL-TP53R172H) on a C57BL/6 background and was kindly provided by Dr. Liwei Wang (Renji Hospital, Shanghai, China). SH-Sgc8c-DBCO (5’-HS-SH C6 -ATC TAA CTG CTG CGC CGC CGG GAA AAT ACT GTA CGG TTA GA-Cy5-TTT TTT TTT -DBCO-3‘), Cy5.5-labeled SH-Sgc8c-DBCO and library ssDNA were synthesized by Sangon Biological Technology Co., Ltd. *Salmonella typhimurium* was purchased from the China General Microbiological Culture Collection Center (CGMCC, China). Plasmid pMD18-luxCDABE and pBBR1MCS2-Tac-GFP and all other reagents were provided by domestic suppliers.

### Growth of bacteria

Attenuated *Salmonella* Typhimurium strain VNP20009 (VNP) carrying plasmid pMD18-luxCDABE and pBBR1MCS2 -Tac-GFP was cultured in LB liquid medium until harvesting at exponential phase (250 rpm, 37 °C). Then, VNP cells were washed by DI water twice and resuspended in DPBS with serial dilution (10, 10^2^, 10^3^, 10^4^, 10^5^ and 10^6^) for counting. The bacterial count was determined by the corresponding optical density (OD at 600 nm) measured by plate reader (BioTek Synergy H1, USA). At the same time, the number of bacteria was determined by counting the colony-forming units (CFUs) after culturing the serial bacterial dilution on selective LB agar plates at 37 °C overnight. Based on this, the standard curve for bacterial CFUs relative to OD values was established as: 1 OD ≈ 2.9 × 10^9^ CFUs/ mL.

### Synthesis of aptamer drug-conjugates (ApDC)

Aptamer drug-conjugates (ApDC) were prepared as previously described.^26^ Briefly, 60 μL of 100 μM DBCO-Sgc8c-SH-SH or DBCO-Library-SH-SH were first reduced by 40 μL of 500 μM TCEP to reduce disulfide. Afterward, 5 μL of 10 mM Vc-MMAE dissolved in DMSO were added in aptamer solution and reacted for 24 h with stirring (400 rpm). Finally, ApDCs (DBCO-Sgc8c-MMAE or DBCO-Lib-MMAE) were purified by HPLC (1260 infinity ii LC system, Agilent Technologies, USA) and characterized by mass spectrometry (Orbitrap Exploris™ 120, Thermo Fisher Scientific, USA).

### Aptamer drug-conjugates (ApDC) anchored on bacterial surface

Azo-dibenzocyclooctyne (DBCO)-functionalized ApDC was anchored on an azide-rich bacterial surface via click chemistry. First, VNP was cultured in liquid LB medium containing 50 μg/mL ampicillin overnight (250 rpm, 37 °C). Then, 1 mL of VNP was diluted into 4 mL of fresh LB medium, and 30 μL of 120 mM 3-azido-D-alanine hydrochloride (Ala-N_3_) were added for another 3–4 h (30 rpm) of culturing, allowing the incorporation of azide groups on the bacterial surface. Next, excess DBCO-functionalized Sgc8c-MMAE or Library-MMAE was added to the VNP-N_3_ suspension after washing twice with ice-cold DPBS and reacting at room temperature overnight. Prepared VNP@ApDC (e.g., VNP@Sgc8c-MMAE, VNP@Lib-MMAE) was purified by centrifugation at 6000 rpm for 3 min.

### Number of Sgc8c-MMAE on bacterial surface

First, a standard curve between fluorescence intensity and the concentration of Cy5-labeled Sgc8c-MMAE was established using a fluorescence spectrophotometer (FluoroMax-4, Horiba, Japan) with excitation at 610 nm and emission at 670 nm. Then, the fluorescence intensity of Cy5-labeled Sgc8c-MMAE on the VNP surface was measured via a fluorescence spectrophotometer, and the molar concentration was calculated using the regression equation of the standard curve. Next, VNP cell number was determined using the spread plate method. The average number of Sgc8c-MMAE molecules per bacterial cell was calculated as

$$N_{avr}=cv \times NA / N$$

*N*_*avr*_ represents the average number of Sgc8c-MMAE on the VNP surface, *c* represents the molar concentration of Sgc8c-MMAE on the VNP surface, *v* represents the volume of VNP@Sgc8c-MMAE for measurement, *NA* is Avogadro’s constant 6.02 × 10^23^ mol^−1^, and *N* represents the number of VNP cells for measurement.

### Characterization of Sgc8c-MMAE-anchored bacteria

To observe the morphological characteristics of VNP@Sgc8c-MMAE, functionalized bacteria were fixed in 2.5% glutaraldehyde at 4 °C for 24 h to stabilize the biofilm. The biofilm was then dehydrated using various ethanol concentrations (30%, 50%, 70%, 80%, 90%, 95%, and 100%) at room temperature for 10 min. Samples were observed using a Scanning Electron Microscopy (SEM) system (JEOL JSM-6310LV, Japan) after undergoing freeze-drying and platinum sputter coating. The zeta potential of native and functionalized bacteria was measured by dynamic light scattering with the standard process (Zetasizer Nano ZS, Malvern Panalytical Ltd., Malvern, UK).

### Stability of ApDC anchored on bacteria

To investigate the serum stability of ApDC on VNP, 1 × 10^9^ CFU VNP@Sgc8c-MMAE labeled with Cy5 was suspended in 5 mL of PBS supplemented with 10% FBS at 37 °C. Designated time points included 0, 2, 4, 8, 24, and 48 h. At certain time point, 100 µL of each sample was retrieved and centrifuged. Following PBS washing, the final samples were resuspended in 100 µL PBS and placed in a quartz cuvette. Fluorescence emission intensity was measured at room temperature with an excitation of 647 nm and an emission of 670 nm. ApDC concentration was calculated using the regression equation obtained from the standard curve.

### Proliferation of bacteria

To examine whether the surface-bound Sgc8c-MMAE affects the growth of VNP, the modified VNP (VNP@Sgc8c-MMAE) was cultured in 100% LB broth. The growth curve over a 12-h period was recorded by monitoring the optical density at 600 nm via plate reader (BioTek Synergy H1, USA).

### Binding ability of VNP@ApDC to pancreatic cancer cells

Cells were seeded in confocal dishes at a density of 2 × 10^5^ cells per dish in 1 mL of DMEM medium containing 10% FBS. Three pancreatic cancer cell lines (Panc-1, Miapaca-2, and KPC1199) were cultured at 37 °C in a 5% CO_2_ environment for 24 h. Subsequently, cells were incubated with Cy5-labeled Sgc8c, Library, Sgc8c-MMAE, or VNP@Sgc8c-MMAE for 30 min at 4 °C. Then cells were washed three times with PBS and visualized using a laser scanning confocal microscope (LSCM, Leica TCS SP8 X, Germany). To quantify cellular uptake, cells were harvested and prepared for flow cytometry analysis.

### Construction of mouse models

Xenograft model: Six-week-old mice were used for xenograft studies. For Miapaca-2 tumor xenograft model, cells were cultured in DMEM supplemented with 10% fetal bovine serum (FBS) and 1% penicillin–streptomycin. For xenograft implantation, 1 × 10^6^ Miapaca-2 cells were resuspended in 100 μL of Matrigel (BD Biosciences) and injected subcutaneously into the right flank of six-week-old nude mice. For KPC1199 tumor xenograft model, cells were cultured in DMEM supplemented with 10% fetal bovine serum (FBS) and 1% penicillin–streptomycin. For xenograft implantation, 3 × 10^5^ KPC1199 cells were resuspended in 100 μL of Matrigel (BD Biosciences) and injected subcutaneously into the right flank of 6-week-old C57 mice. Tumor volume was measured using a digital caliper and calculated as volume=width^2^ × length × 0.5. Experiments were conducted when the tumor volume reached 100 mm^3^.

PDX model was kindly provided by Liwei Wang and his group members (KY [2019] 035).

In-situ carcinoma model: A total of 2 × 10^5^ KPC1199-Luc cells resuspended in 10 μL of Matrigel were implanted into the pancreatic epithelium of C57 mice (Male, 6-week-old). Subsequently, the fluorescence signal was captured with an IVIS Lumina XP imaging system. Experiments were conducted when the tumor volume reached 100 mm^3^.

All studies were reviewed and approved by the Ethics Committee of Renji Hospital (RJ2021-0621) and were performed according to NIH guidelines.

### Flow cytometry analysis

Tumors were excised, minced, and digested in collagenase IV (1 mg/mL) and DNase I (0.1 mg/mL) for 30 minutes at 37 °C. Single-cell suspensions were prepared by filtering through a 70-μm cell strainer. Cells were stained with antibodies against CD45, CD4, CD8, CD11c, MHCII, Ly6G, F4/80, IFN-γ, TNF-α, T-bet, and granzyme B (GzmB). Data were acquired on a flow cytometer and analyzed using FlowJo software.

### Biodistribution study

To investigate biodistribution, mice bearing Miapaca-2 or KPC1199 tumors were intravenously administered with 0.1 mL of VNP, VNP@Lib-MMAE, and VNP@Sgc8c-MMAE containing 1 × 10^7^ CFU (*n* = 3 mice/group). All mice were imaged at certain time points with an IVIS Lumina XP imaging system. Main organs and tumors were collected after imaging and then homogenized in a glass homogenizer. The homogenates were diluted in LB medium, and 50 μL of each dilution was plated on LB agar plates containing antibiotics. The plates were then incubated overnight at 37 °C for bacterial colony counting.

### Calculation of distance between ApDC and tumor edge

Tumor tissue images underwent processing in both ImageJ and MATLAB to generate a semi-quantitative Euclidean Map, following a methodology similar to that described by Ian Nessler et al.^39^ Briefly, the 405 nm plane was isolated and transformed into a binary image, effectively masking all areas within the tumor tissue, with only pixels outside the tumor tissue retaining non-zero intensity. This binary image was then exported to MATLAB and processed using the built-in Euclidean Distance Transform function, resulting in a pixel distance matrix. This matrix contained the distance of each pixel within the tumor from the nearest non-zero pixel, predominantly situated around the edges of the tumor tissue. Next, the 488 nm and 635 nm planes of the same image were isolated and converted into grayscale images by MATLAB, where the signal intensity at each pixel was extracted and plotted against the corresponding Euclidean distance. This process yielded a Euclidean distance map illustrating the spatial distribution of signal intensity within the tumor tissue.

### Single-cell sequencing

Single-cell Isolation and Sequencing Library Preparation: Tumor samples from mice treated with KPC1199 were processed into single-cell suspensions. Post-quality assessment, the cell concentration was set between 700 and 1200 cells per microliter for compatibility with the 10x Genomics Chromium™ system. The mRNA underwent reverse transcription using a poly(T) primer that incorporates a barcode sequence via the Template Switch Oligo (TSO).

scRNA-seq: Gene expression matrix was generated per sample using CellRanger (v6.1.2) and then converted to a Seurat object using the Seurat R package (v4.0.4). Predicted doublets were removed using DoubletFinder (v2.0.3). Batch effect was performed on each sample using the R package harmony (v0.1.0). Cell type annotation was based on the expression of biomarkers in each cluster. Tumor cells were reconfirmed using the R package infercnv (v1.12.0).

### In vivo cancer therapy

Mice were divided into different groups, and PBS, VNP, VNP@Sgc8c-MMAE or other related drugs (100 µL) were injected intravenously. Tumor dimensions were measured every other day with digital calipers.

### ELISA assay for cytokine secretion

Tumor tissues were collected and homogenized after euthanizing the mice on day 14. The concentrations of cytokines (TNF-α, IFN-γ, CCL-5 and IL-6) in the tumor tissues were then measured using ELISA following the manufacturer’s protocol.

### H&E staining

Tumors and main organs were collected from mice and fixed in 4% paraformaldehyde. H&E staining was performed according to the manufacturer’s protocol.

### Immunohistochemistry

Tumor tissues were fixed in 10% neutral buffered formalin, embedded in paraffin, and sectioned at 4–5 μm thickness. Sections were deparaffinized, rehydrated, and subjected to antigen retrieval. Endogenous peroxidase activity was blocked, followed by incubation with primary antibodies against Ki67, α-SMA, PTK7, and collagen. After washing, sections were incubated with secondary antibodies, followed by streptavidin-HRP and DAB substrate for color development. Then sections were scanned with the Digital Pathology Slide Scanner (KF-PRO-120, China).

### Masson’s trichrome staining

Sections were stained to differentiate collagen fibers, with collagen fibers staining blue, muscle fibers red, and nuclei black. The extent of fibrosis was quantified using image analysis software.

### TUNEL assay

Tissues were fixed in 4% paraformaldehyde for 1 h at room temperature and then treated with 0.5% TritonX-100 for 10 min. After washing with PBS, cells were incubated with the cell death detection kit (Roche, Basel, Switzerland), according to the manufacturer’s instructions. Nuclei were costained for 5 min with 0.1 g/mL DAPI (Beyotime, Nantong, China), and images were captured under a microscope (Olympus, BX53; Melville, NY, USA).

### Statistical analysis

All data are presented as mean ± standard deviation. Statistical analysis between two groups was performed using ANOVA analysis followed by Fisher’s LSD multiple comparison. A *P* value of <0.05 was considered statistically significant. *n* = 3 to 8; mean ± standard deviation.

## Supplementary information

Supplementary Material

## Data Availability

The dataset resulting from the present study is accessible through the corresponding authors upon submission of a reasonable request. The single-cell sequencing data supporting the findings of this study have been deposited in the Gene Expression Omnibus (GEO) database under accession number GSE276051.
