# Supplementary material for: Aptamer-drug conjugates-loaded bacteria for pancreatic cancer synergistic therapy
Source: Signal Transduct Target Ther. 2024 Oct 14;9:272. doi: 10.1038/s41392-024-01973-3 (PMC11471780; doi:10.1038/s41392-024-01973-3)
Supplement: Supplementary file 1 — Supplementary Material [file 41392_2024_1973_MOESM1_ESM.docx]

Supplementary Materials for

Aptamer-drug conjugates-loaded bacteria for pancreatic cancer synergistic therapy

Yu Xiao ^1^, Tao Pan ^1^, Wuren Da ^1^, Yuanding Liu ^1^, Shuangya Chen ^1^, Daiquan Chen ^1^, Keying Liu ^1^, Yihan Zheng ^1^, Daolong Xie ^1^, Yuan Gao ^1^, Haiyan Xu ^1^ ✉, Yang Sun ^1^ ✉ and Weihong Tan ^1,2^ ✉

✉Correspondence:

Haiyan Xu, uhaiyan@sjtu.edu.cn;

Yang Sun, [suny1989@sjtu.edu.cn](mailto:suny1989@sjtu.edu.cn);

Weihong Tan, tan@hnu.edu.cn.

**This PDF file includes**:

Supplementary Fig. 1 to Supplementary Fig. 15


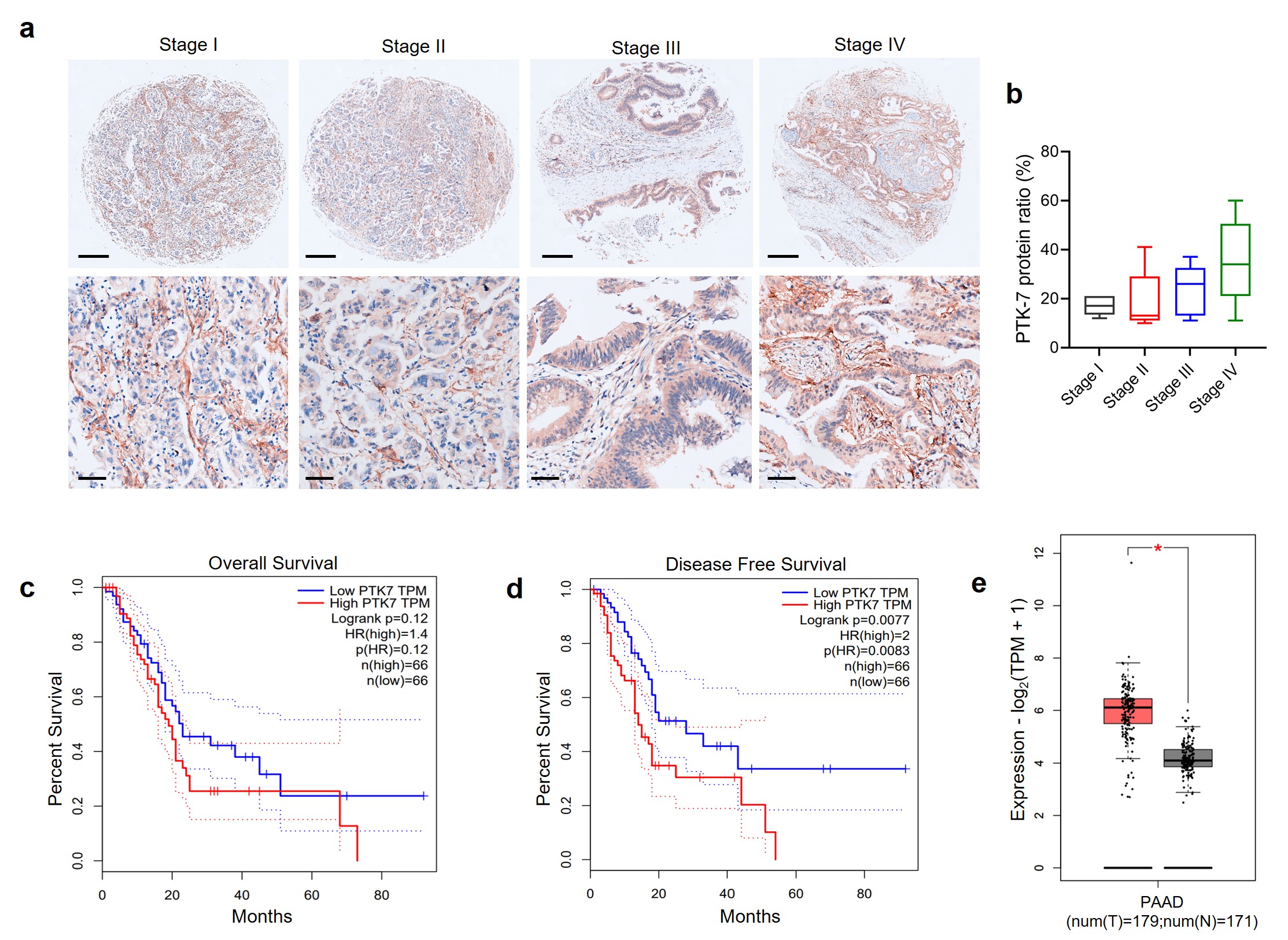


Supplementary Fig. 1

**Analysis of PTK7 expression in pancreatic tumor tissues and its correlation with survival outcomes. (a)** Immunohistochemical staining of PTK7 protein in tumor tissue samples from Stage I to Stage IV. The upper panel shows low magnification images, while the lower panel shows higher magnification images. Scale bars, upper panel: 250 µm; lower panel: 50 µm. **(b)** Quantification of PTK7 protein expression levels in tumor tissues from different cancer stages. Data are presented as mean ± standard deviation. The expression of PTK7 increases progressively from Stage I to Stage IV, with the highest levels observed in Stage IV. **(c)** Kaplan-Meier analysis of overall survival in patients with low (blue) and high (red) PTK7 transcript per million (TPM) expression levels. Log-rank p=0.12, HR (high)=1.4. **(d)** Kaplan-Meier analysis of disease-free survival in patients with low (blue) and high (red) PTK7 TPM expression levels. Log-rank p=0.0077, HR (high)=2. n(high)=66, n(low)=66. **(e)** Box plot showing PTK7 expression levels in pancreatic adenocarcinoma (PAAD) tissues.


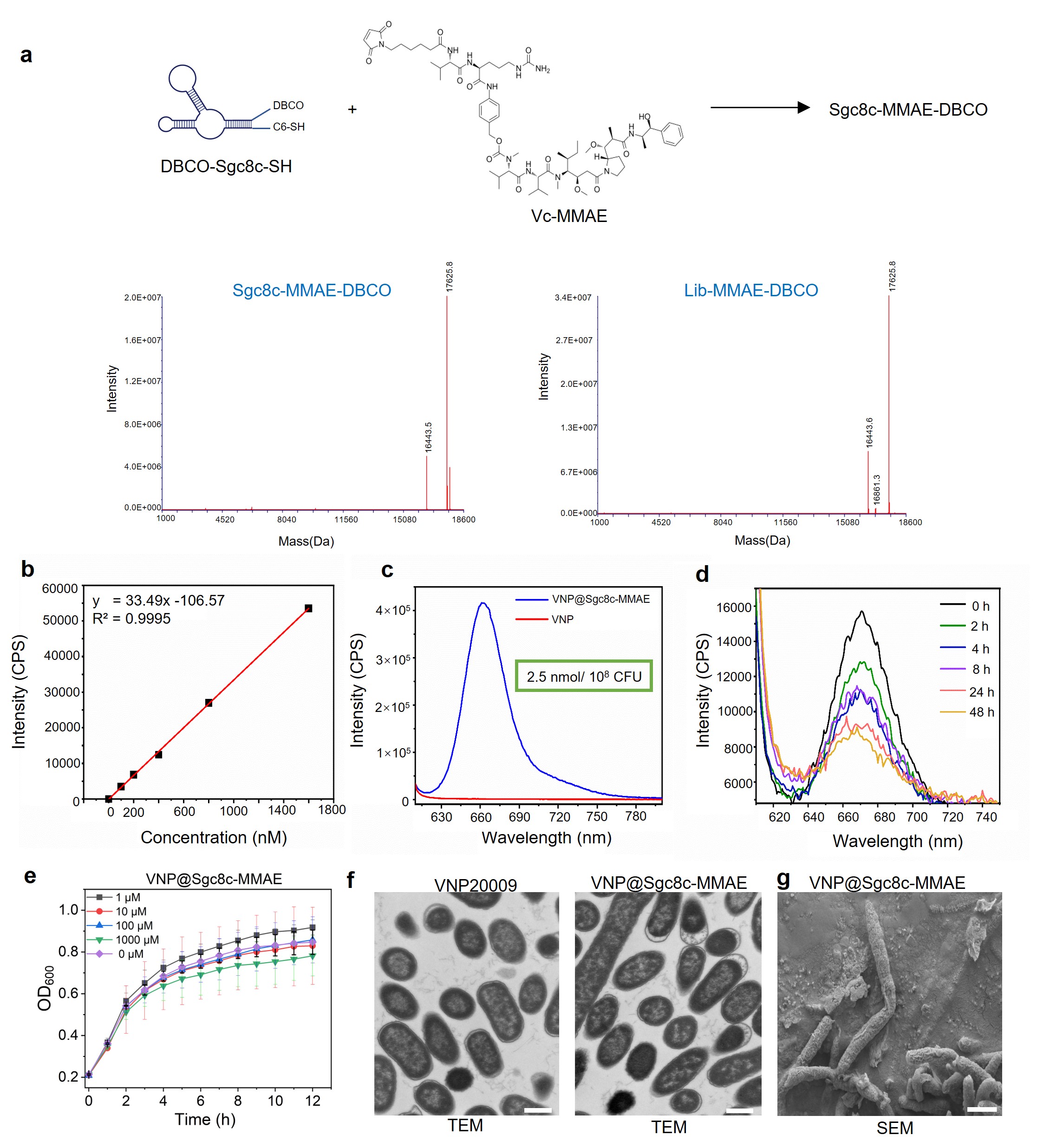


Supplementary Fig. 2

**Characterization of VNP@Sgc8c-MMAE. (a)** Schematic diagram for synthesis process of ApDC-DBCO with DBCO-Sgc8c-SH and Vc-MMAE. Mass spectrometry (MS) analysis of Sgc8c-MMAE-DBCO and Lib-MMAE-DBCO performed by Sangon Biotech (Shanghai). The calculated molecular weight was 17625 for Sgc8c-MMAE-DBCO and 17626 for Lib-MMAE-DBCO. The observed RNA peak was 17625.8 for Sgc8c-MMAE-DBCO and 17625.8 for Lib-MMAE-DBCO. **(b)** Standard curve of Cy5-Sgc8c-MMAE at different concentration detected by fluorescence spectrophotometry. **(c)** Fluorescence intensity of VNP and VNP@Sgc8c-MMAE-Cy5. The content of Sgc8c-MMAE-Cy5 coupled on the bacterial surface was calculated based on the standard curve. **(d)** Fluorescence intensity of Sgc8c-MMAE-Cy5 on the bacterial surface after incubating with 20% FBS. **(e)** Growth curves of VNP@Sgc8c-MMAE anchored with different concentrations of Sgc8c-MMAE (0-1000 μM). OD600 was recorded at 1 h intervals by microplate reader. Results are shown as mean ± SD of three independent experiments. **(f)** Transmission electron microscopy images (TEM) of VNP20009 and VNP@Sgc8c-MMAE. Scale bar: 1 μm. **(g)** Scanning electron microscopy (SEM) image of VNP@Sgc8c-MMAE. Scale bar: 1 μm.


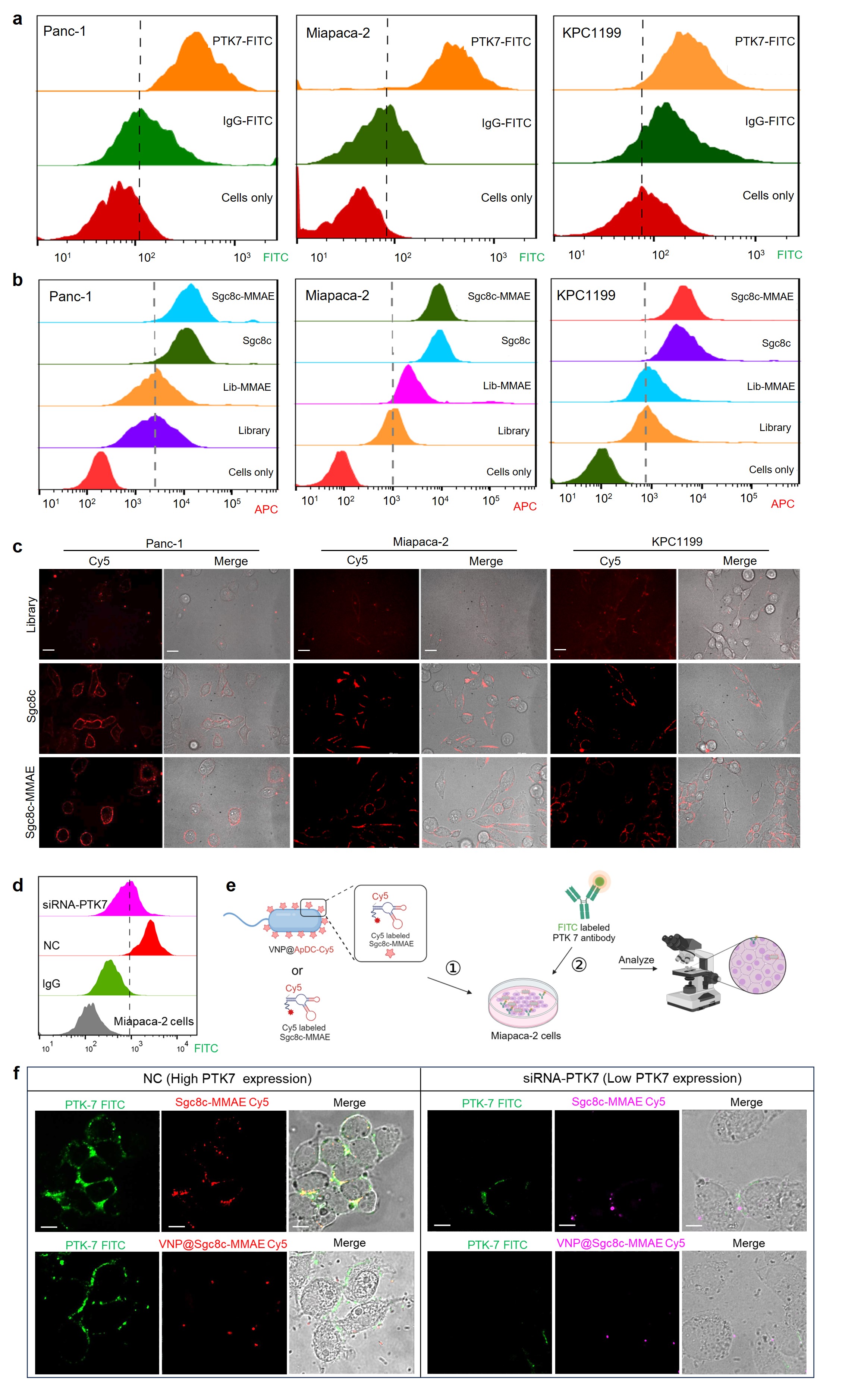


Supplementary Fig. 3

**Binding ability of ApDC with pancreatic cancer cells. (a)** Flow cytometric analysis of PTK-7 receptors expressing on Panc-1, Miapaca-2 or KPC1199 cells. Cells was incubated with FITC-labeled PTK7 antibody on ice for 30 min with shaking at 100 rpm. **(b)** Flow cytometric analysis of Cy5-labeled aptamer or ApDC binding on pancreatic cancer cells (Panc-1, Miapaca-2 or KPC1199). Cells pellets was incubated with 250 nM aptamer or ApDC for 30 min above ice with shaking. **(c)** LSCM images of cell binding ability of Cy5-labeled Sgc8c, Library and Sgc8c-MMAE against Panc-1, Miapaca-2 or KPC1199 cells. Scale bar: 25 μm. **(d)** Flow cytometry analysis of PTK-7 expression levels in Miapaca-2 cells. Fluorescence intensity was detected using FITC-labeled PTK-7 antibodies for cells with high and low PTK-7 expression. Image created with Biorender.com, with permission. **(e)** Schematic illustration of Sgc8c-MMAE and VNP@Sgc8c-MMAE-Cy5 labeling for PTK-7. Sgc8c-MMAE was labeled with Cy5 and used to bind PTK-7 high and low expression Miapaca-2 cells. **(f)** Immunofluorescence microscopy analysis of PTK-7 high and low expression Miapaca-2 cells. The left column shows fluorescence images of PTK-7 FITC labeling, the center column shows fluorescence images of Sgc8c-MMAE Cy5 labeling, and the right column shows merged images. Scale bar: 10 μm.


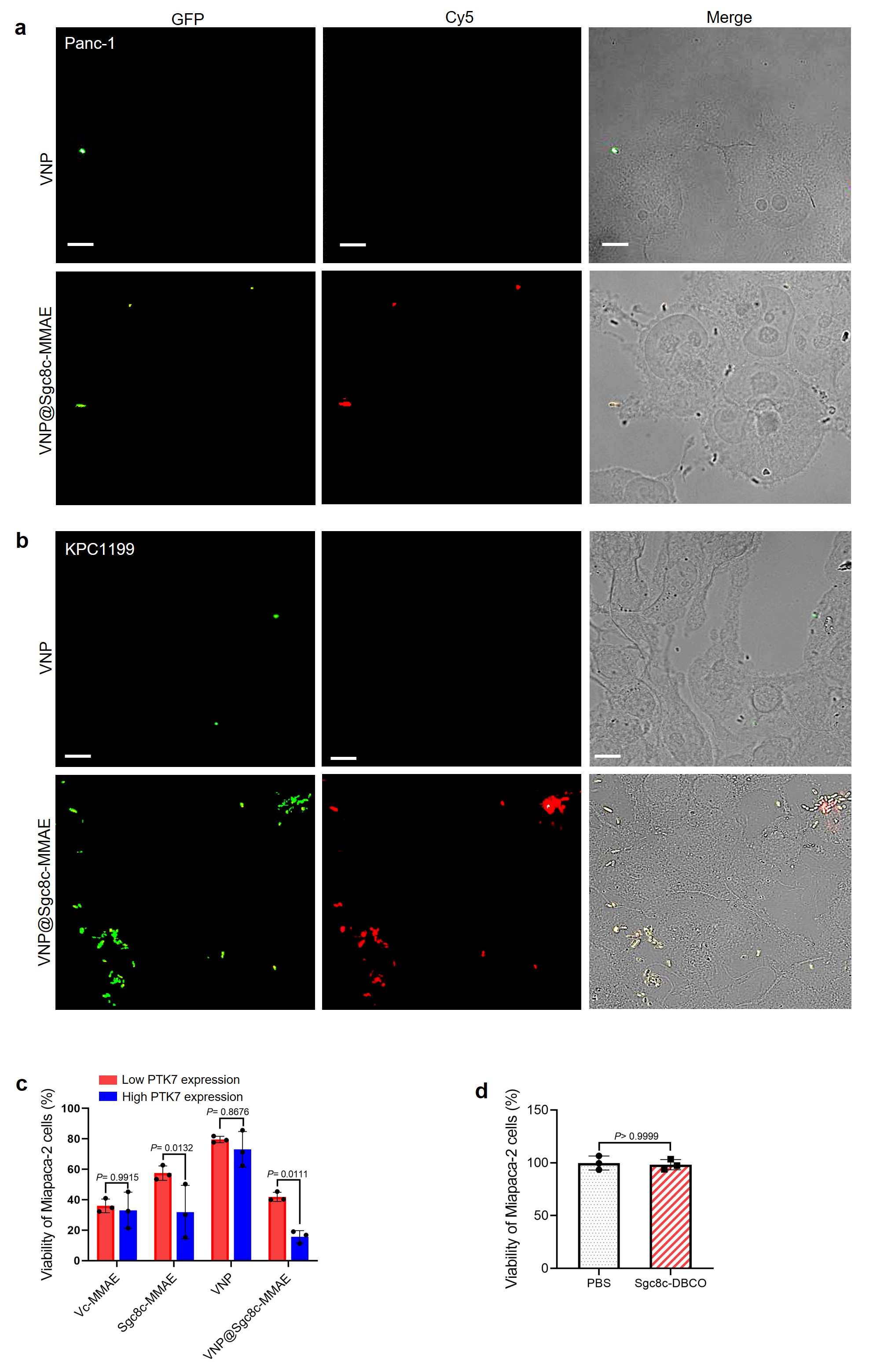


Supplementary Fig. 4

**Binding ability of VNP@Sgc8c-MMAE to pancreatic cancer cells.** Laser scanning confocal microscopy images of VNP_GFP_ and VNP@Sgc8c-MMAE incubated with **(a)** Panc-1 or **(b)** KPC1199 cells for 2 h at 37℃. Green channel shows VNP_GFP_, and red channel represents Cy5-labeled Sgc8c-MMAE anchored on VNP_GFP_. Scale bar: 10 μm. **(c)** Viability of Miapaca-2 cells with different PTK-7 protein level treated with VNP20009, Sgc8c-MMAE, Vc-MMAE or VNP@Sgc8c-MMAE at equal Vc-MMAE concentration (256 nM) for 72 h. VNP20009 concentration:10^7^ CFU. **(d)** Viability of Miapaca-2 cells treated with DBCO labeled Sgc8c (concentration: 256 nM) for 72 h. Data are shown as mean±s.d. of 3 independent experiments.


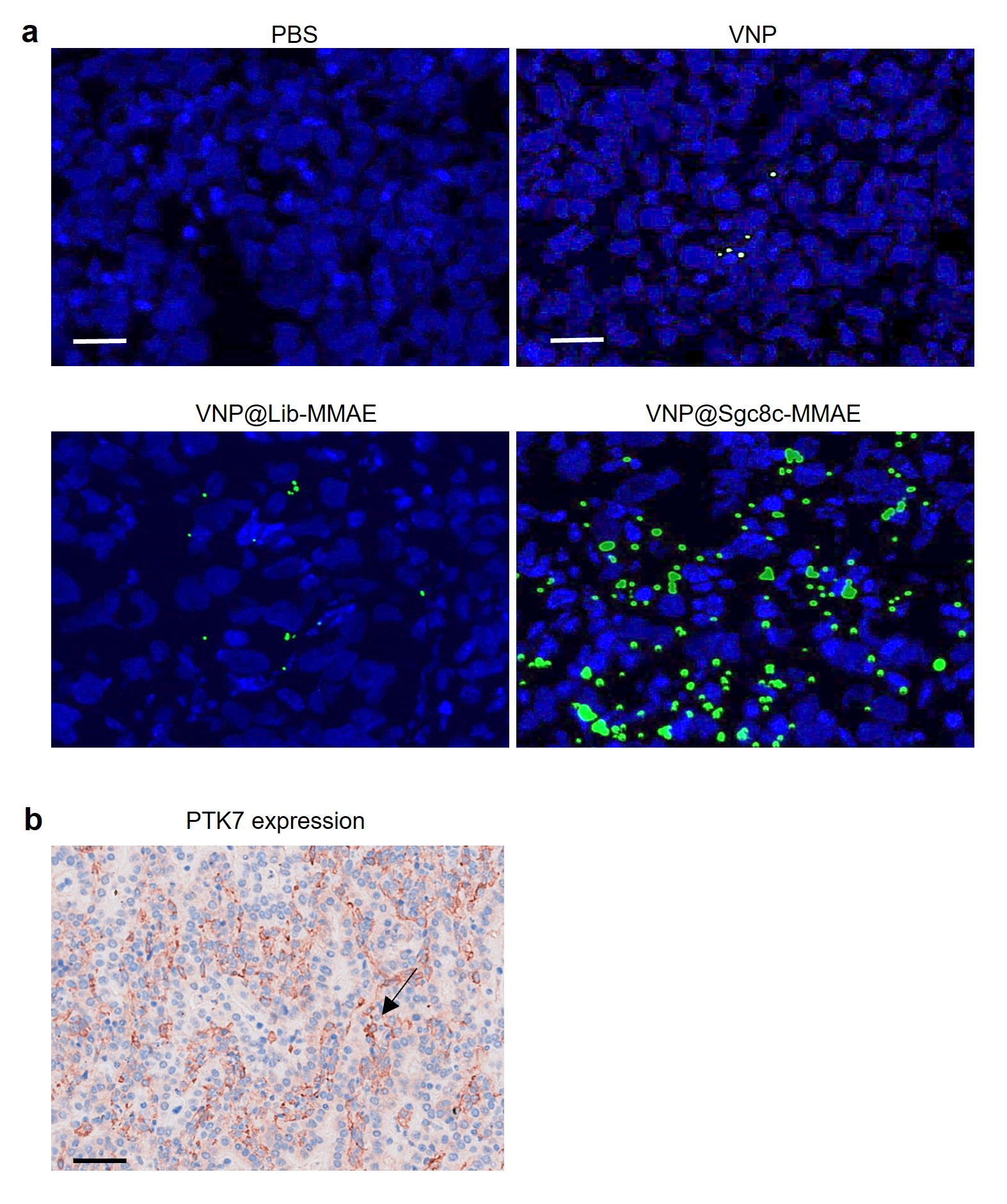


Supplementary Fig. 5

**Colonization of ApDC anchored VNP in pancreatic tumor tissue. (a)** Representative confocal fluorescence images of Miapaca-2 subcutaneous tumor tissues sectioned at 24 h after i.v. injection of VNP_GFP_. The green and blue channels indicate VNP_GFP_ and nuclei stained with DAPI respectively. Scale bar: 20 μm. **(b)** Immunohistochemical analysis of PTK7 expression in Miapaca-2 tumor tissue. Arrows indicate PTK7-positive cells. Scale bars represent 50 μm.


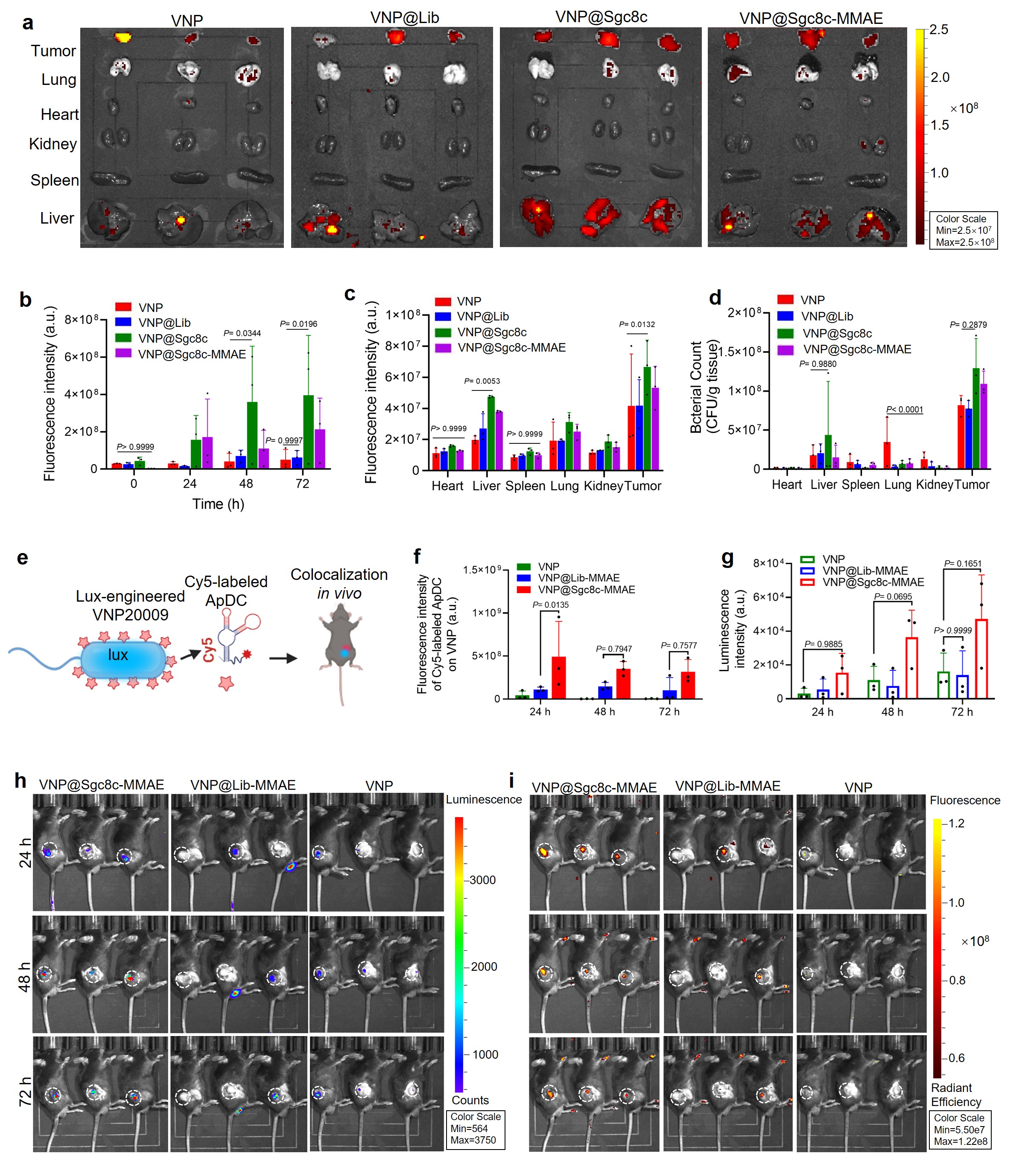


Supplementary Fig. 6

**Targeting enrichment and colocalization of VNP@Sgc8c-MMAE at tumor site.** Miapaca-2 tumor-bearing nude mice were i.v. injected with 10^7^ CFUs of GFP-expressing VNP, VNP@Lib, VNP@Sgc8c or VNP@Sgc8c-MMAE for 72 hours, main organs and tumor tissue were collected for *ex vivo* imaging. **(a)** *Ex vivo* IVIS fluorescence images of major organs and tumors from Miapaca-2 tumor-bearing mice. **(b)** Fluorescence intensity from tumor region at 24, 48 and 72 h calculated by IVIS Lumina II system. **(c)** Fluorescence intensity of major organs and tumors in Miapaca-2-bearing mice after i.v. injection of bacterial preparations for 72 h. **(d)** Bacterial counts of VNP, VNP@Lib, VNP@Sgc8c, and VNP@Sgc8c-MMAE in tumor and major tissues 72 hours after the first treatment. Data are presented as mean ± standard deviation (n = 3) and were analyzed using a two-way ANOVA, followed by Fisher's LSD multiple comparisons test. **(e)** To colocalize Lux-expressing VNP and Cy5-labeled ApDC *in vivo*, KPC1199 tumor-bearing C57 mice were i.v. injected with 10^7^ CFUs of Lux-expressing VNP, Lux-expressing, and Cy5-labeled VNP@Lib-MMAE, or VNP@Sgc8c-MMAE for 72 hours, schematic illustrating the construction of functionalized bacteria and the colocalization investigation *in vivo*. Image created with Biorender.com, with permission. **(f)** Fluorescence intensity of Cy5-labeled ApDC from tumor region at 24, 48 and 72 h calculated by IVIS Lumina II system. **(g)** Luminescence intensity of bacteria from tumor region at 24, 48 and 72 h calculated by IVIS Lumina II system. **(h)** IVIS Lumina images of major organs and tumors from KPC1199 tumor-bearing mice to map bacterial distribution *in vivo*. **(i)** IVIS fluorescence images of major organs and tumors from KPC1199 tumor-bearing mice to map ApDC distribution *in vivo*.


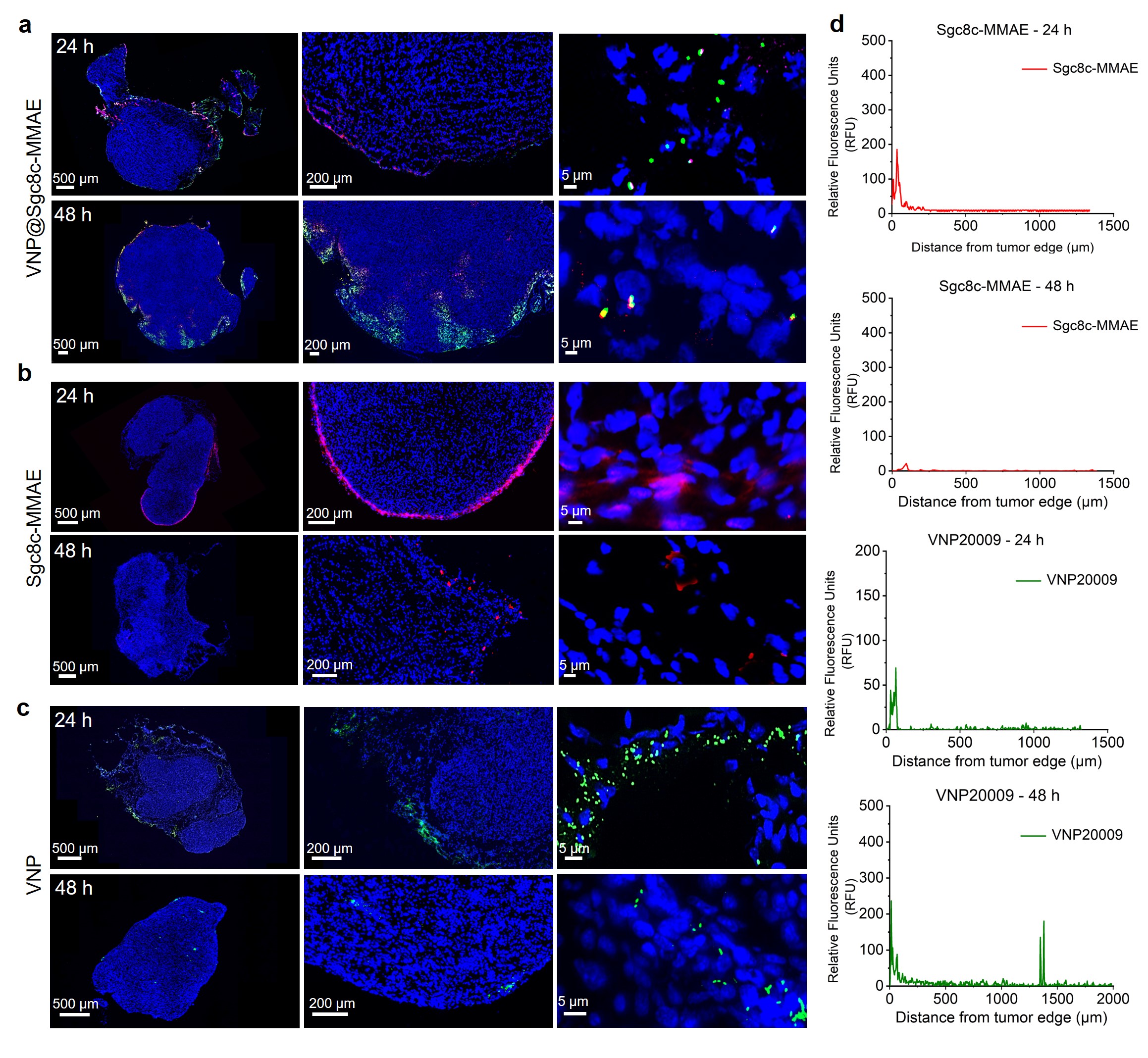


Supplementary Fig. 7

**Dynamic visualization of VNP@Sgc8c-MMAE or Sgc8c-MMAE in KPC1199 subcutaneous tumor tissues.** Confocal images of tumor tissue sections collected from KPC1199 subcutaneous tumor- bearing mouse at 24 h and 48 h post-intravenous injection of **(a)** VNP@Sgc8c-MMAE or **(b)** Cy5-labeled Sgc8c-MMAE or **(c)** VNP20009. Nuclei stained with DAPI are shown in blue, and Cy5-labeled ApDC appear in violet. **(d)** Quantification of payload penetration. The green represents VNP20009 and violet represents Cy5-labeled Sgc8c-MMAE.


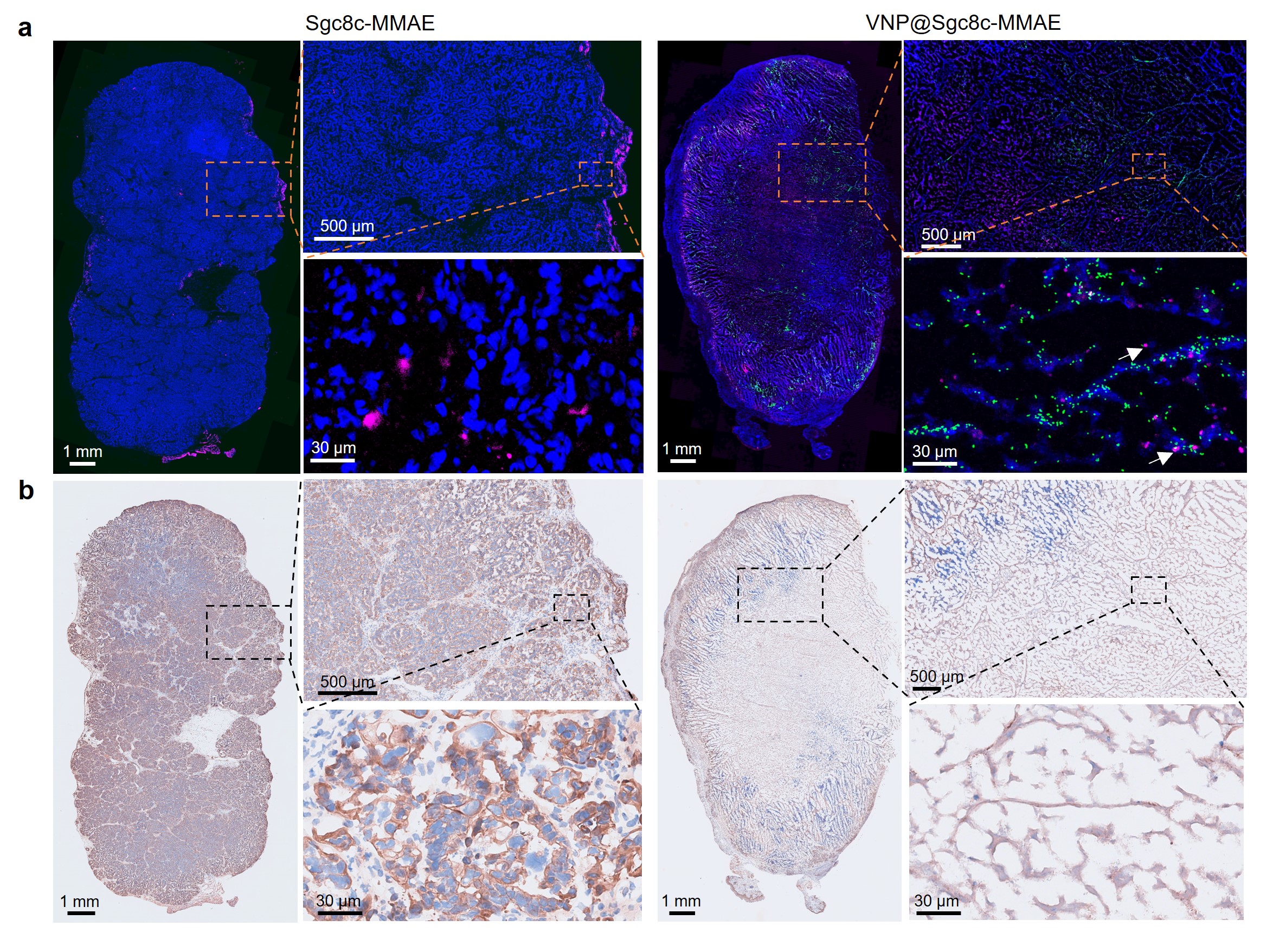


Supplementary Fig. 8

**Penetration behavior of VNP@Sgc8c-MMAE or Sgc8c-MMAE in pancreatic cancer PDX mouse model. (a)** Confocal images of tissue sections collected from the PDX mouse model after i.v. injection of Sgc8c-MMAE-Cy5 or VNP@Sgc8c-MMAE-Cy5 for 48 h. VNP_GFP_ is represented in green, cell nuclei are depicted in blue, and Cy5-labeled ApDC is shown in violet. **(b)** The distribution of α-SMA (alpha-smooth muscle actin) in tumor tissues visualized after staining with a mouse monoclonal anti-α-SMA antibody. The orange or black dashed box marks the area selected for obtaining the high-resolution image.


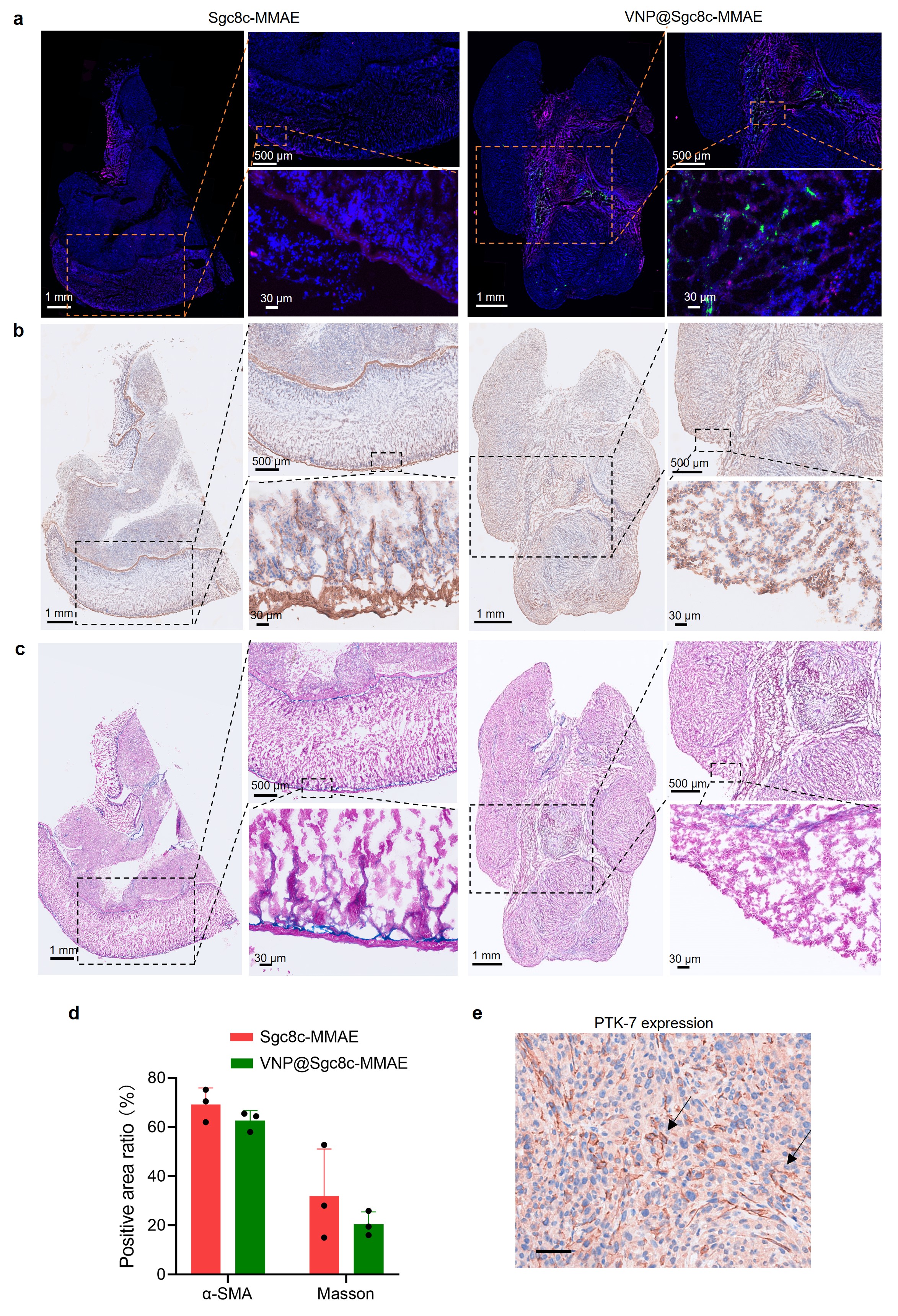


Supplementary Fig. 9

**Penetration behavior of VNP@Sgc8c-MMAE or Sgc8c-MMAE in the KPC1199 pancreatic *in situ* mouse model. (a)** Confocal images of tissue sections collected from the *in-situ* carcinoma pancreatic cancer model post-administration of Sgc8c-MMAE-Cy5 or VNP@Sgc8c-MMAE-Cy5 for 48 h. Sections were prepared and visualized with confocal microscopy, where VNP_GFP_ appeared green, DAPI-stained cell nuclei appeared blue, and Cy5 labeled Sgc8c-MMAE appeared violet. **(b)** The distribution of α-SMA in tumor tissues visualized after staining with a mouse monoclonal anti-α-SMA antibody. **(c)** Masson's trichrome staining of the tumor tissues showing the extent of fibrosis within the tumors. The VNP@Sgc8c-MMAE group demonstrates a decrease in fibrotic areas compared to the Sgc8c-MMAE group. **(d)** Quantification of the positive area ratios for α-SMA and Masson’s trichrome staining. **(e)** Immunohistochemical analysis of PTK7 expression in KPC1199 tumor tissue. Arrows indicate PTK7-positive cells. Scale bars represent 50 μm. The orange or black dashed box marks the area selected for obtaining the high-resolution image.


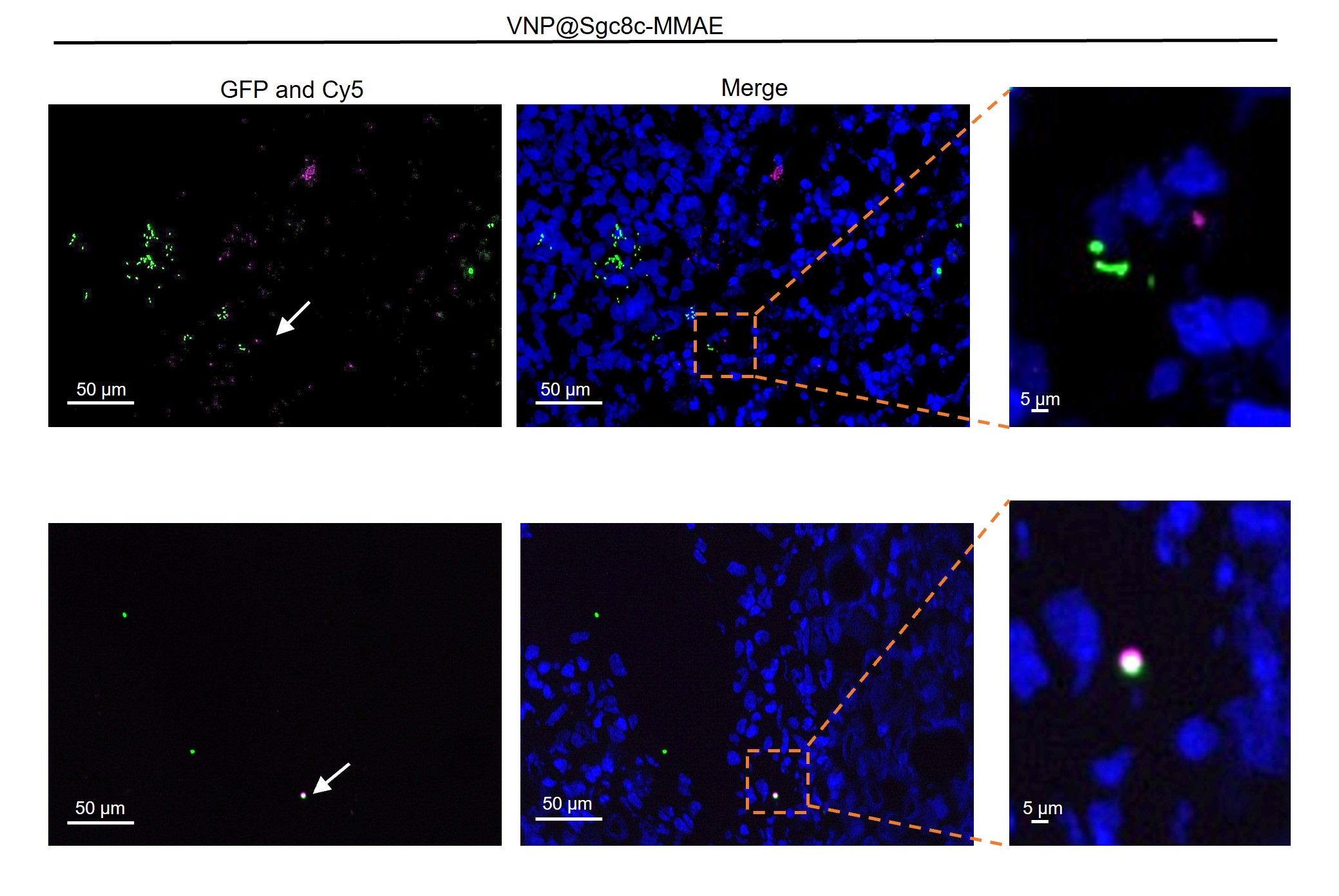


Supplementary Fig. 10

**Release behavior of Sgc8c-MMAE from bacterial surfaces.** Confocal images of tumor tissue sections collected from the Miapaca-2 tumor-bearing mice post-administration of Sgc8c-MMAE-Cy5 or VNP@Sgc8c-MMAE-Cy5 for 72 h. Fluorescence imaging was performed to examine the release of Cy5-labeled Sgc8c-MMAE (violet) from the surface of VNP_GFP_ (green) within the tumor tissue sections. The orange box marks the area selected for obtaining the high-magnification field image.


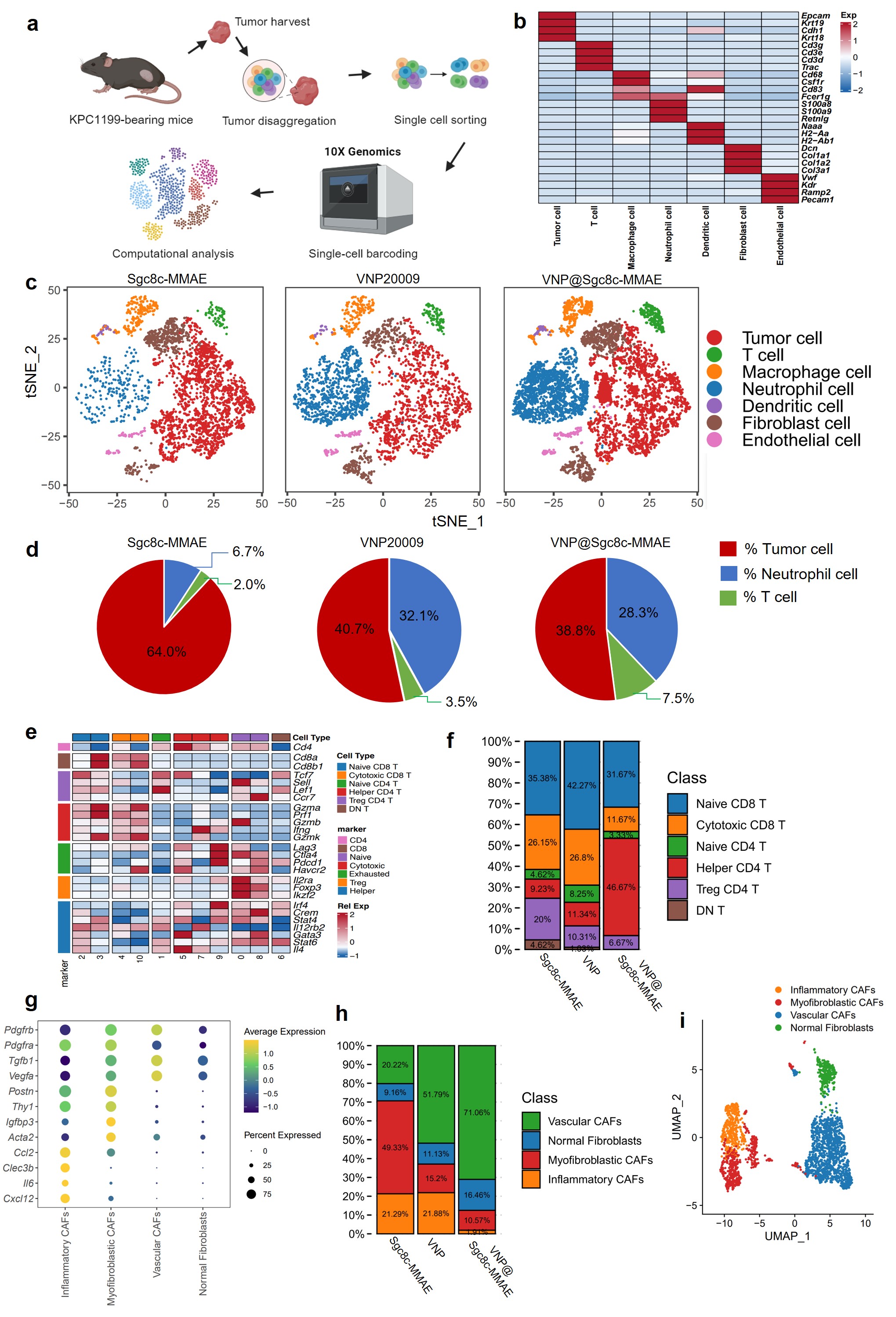


Supplementary Fig. 11

**Single-cell sequencing analysis of tumors from the KPC1199 subcutaneous tumor model after multiple dosing.** **(a)** Diagram illustrating the single-cell sequencing process. Image created with Biorender.com, with permission. **(b)** Heatmap plot demonstrating the relative expression of marker genes in the indicated cell populations **(c)** T-SNE plots showing clusters of intra-tumoral cell populations in PDAC samples. In the plot, the X and Y axes signify reduced-dimensional components. Each dot represents an individual cell with proximity indicating similarity in cell type. Distinct cell groupings are highlighted in varied colors. **(d)** The proportions of various cell types, including tumor cells, neutrophils, and T cell clusters, in the Sgc8c-MMAE, VNP20009, and VNP@Sgc8c-MMAE groups. **(e)** Heatmap plot showing the canonical marker genes across all T cells. **(f)** Histogram plot showing the T cells composition percentage of each group. **(g)** Dot plot showing the expression levels of canonical marker genes across all fibroblasts. **(h)** Histogram plot showing the fibroblasts composition percentage. **(i)** UMAP plot showing the cell types identified from fibroblast. UMAP: uniform manifold approximation and projection.


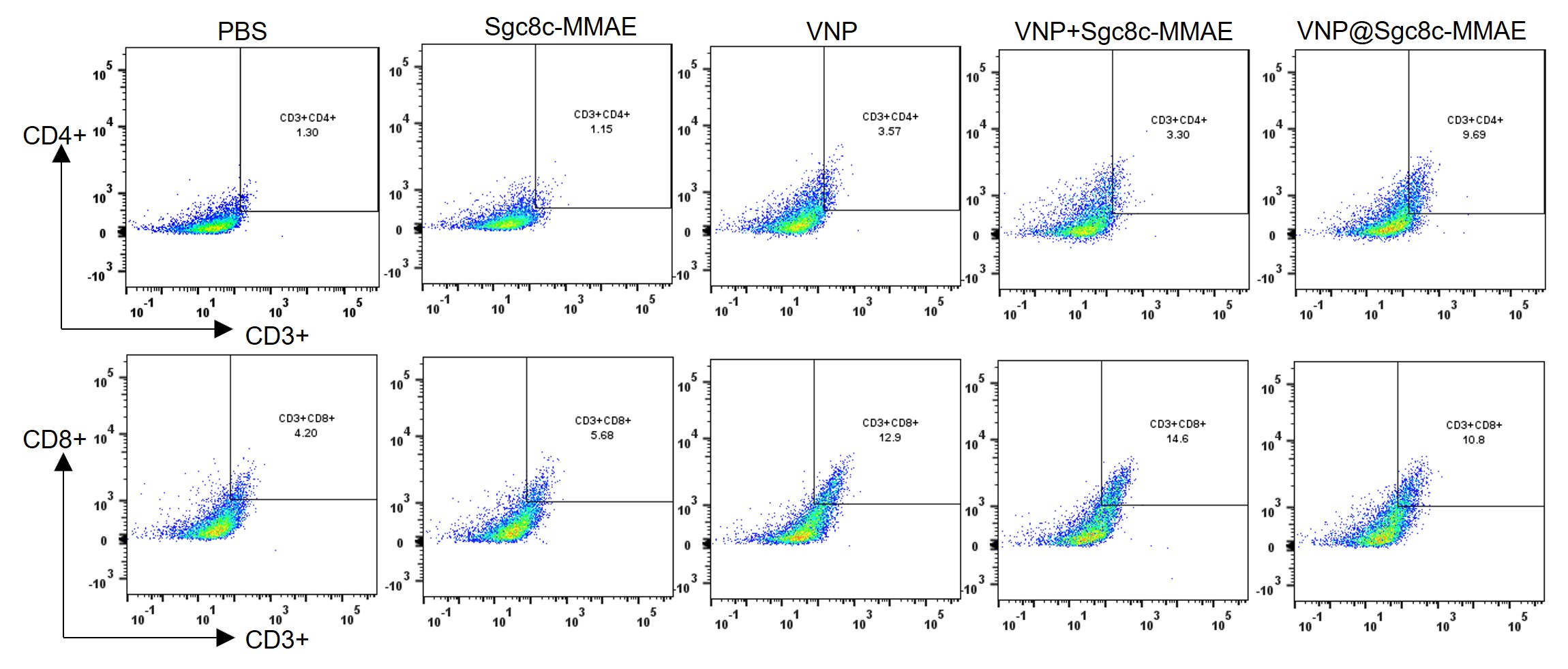
Supplementary Fig. 12

**VNP@Sgc8c-MMAE upregulates the proportion of intratumoral T cells.** Flow cytometric plot of T cells in KPC1199 subcutaneous tumor model post-multiple interventions. The tissues were digested into single cells with collagenase and the proportion of CD4^+^ and CD8^+^ cells gated on CD3 T cells within tumor tissues were studied with anti-CD4, anti-CD8 and anti-CD3 antibody using flow cytometry.


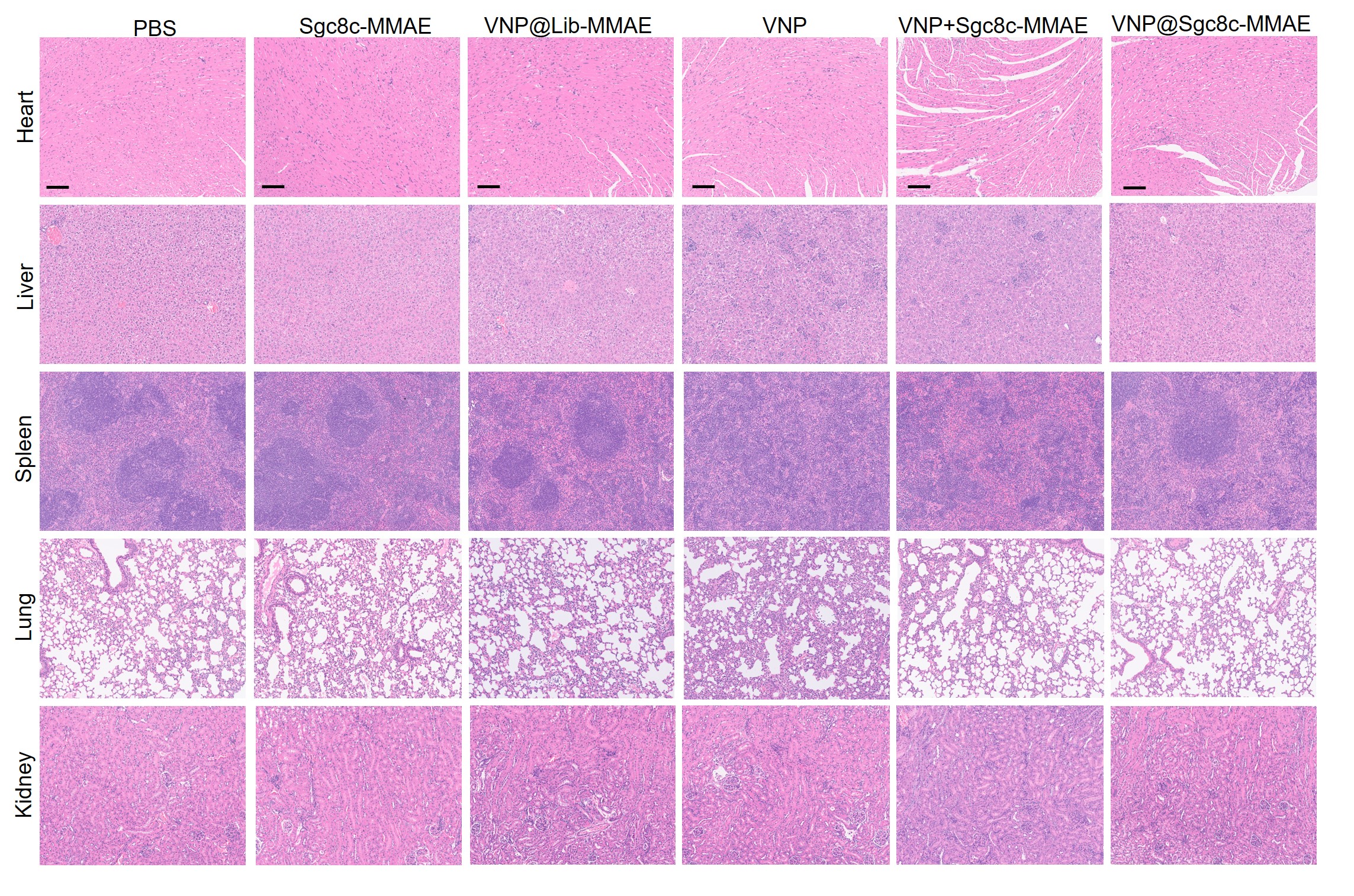


Supplementary Fig. 13

**H&E staining of main organs from KPC1199 subcutaneous tumor mode.** Main organs of KPC1199 subcutaneous tumor model were precisely harvested and subjected to an exhaustive histological preparation process, encompassing fixation, slicing, and staining with hematoxylin and eosin (H&E). Scale bar: 100 μm.


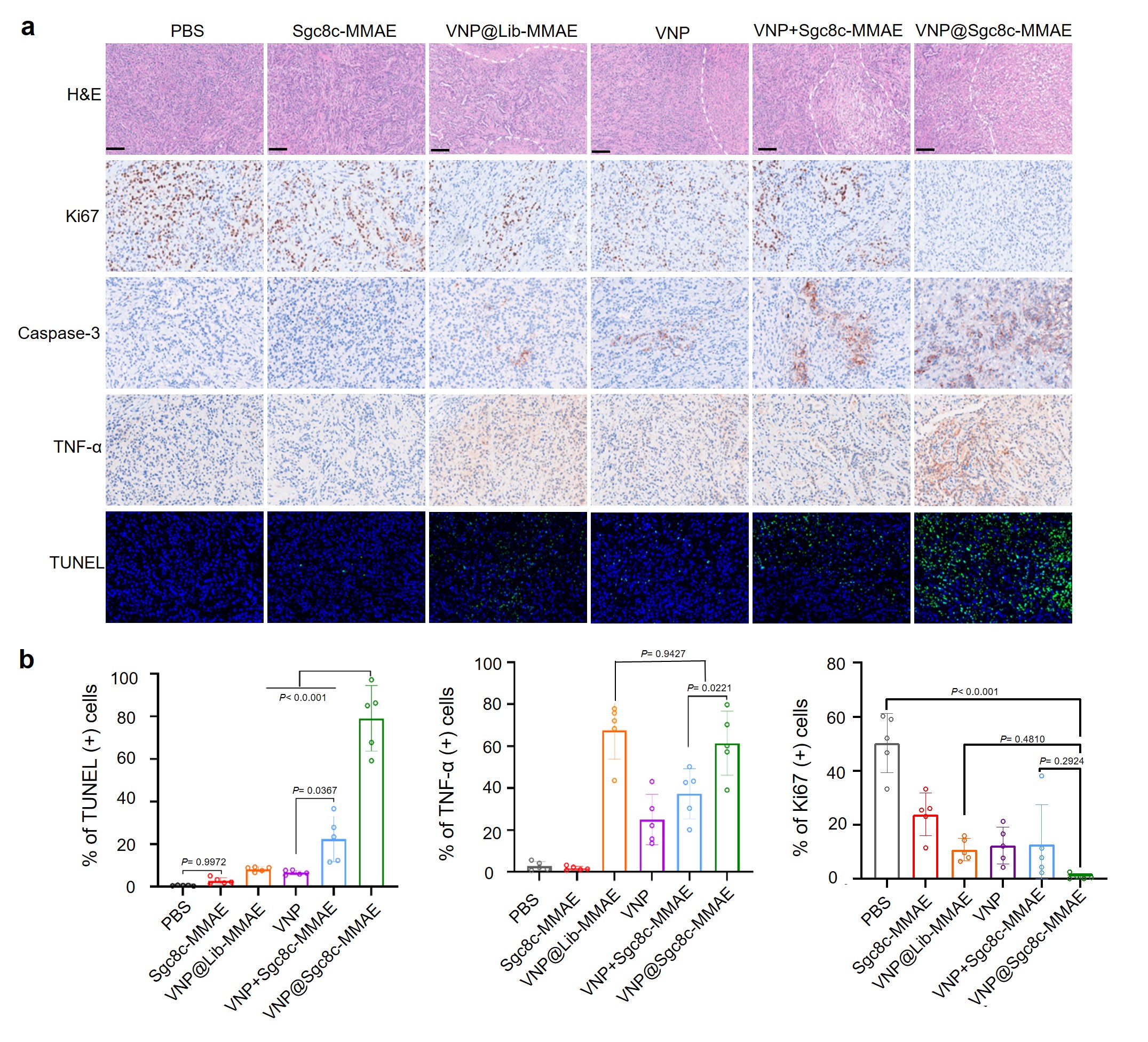


Supplementary Fig. 14

**IHC and IF images and statistical analysis of tumor tissues in the KPC1199 subcutaneous tumor model. (a)** Immunohistochemical (H&E, Ki67, caspase-3 and TNF-α) and TUNEL staining of tumor tissues from KPC1199 subcutaneous tumor models with different treatments. **(b)** Proportions of positive cells for TUNEL, TNF-α, and Ki67 staining. Five fields of view were randomly selected to count the proportions of cells positive for each group of sections. Data are presented as mean ± s.d. (n = 5 mice per group, one-way ANOVA analysis followed by Fisher’s LSD multiple comparison). Scale bar: 100 μm.


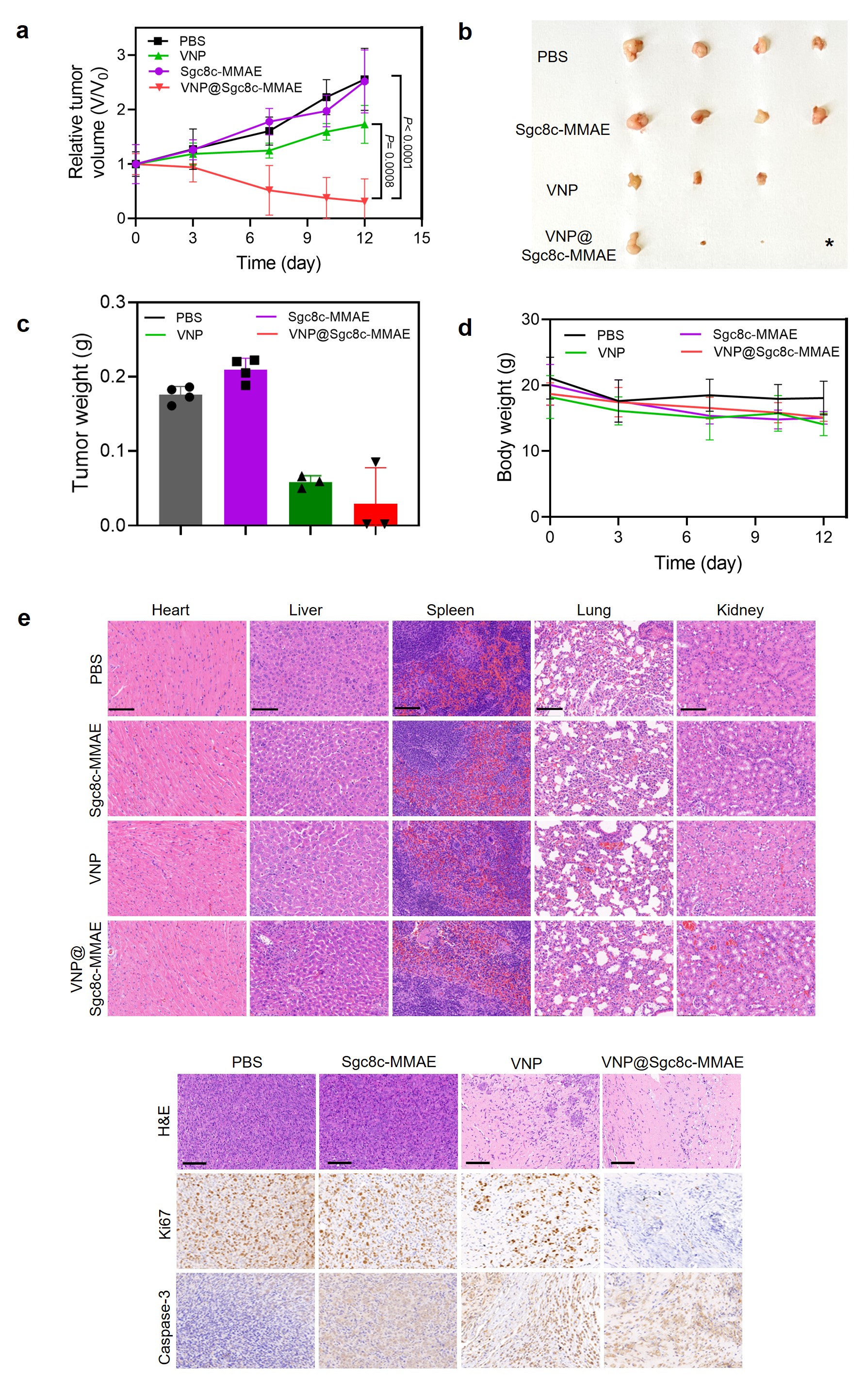


Supplementary Fig. 15

***In vivo* therapeutic efficacy of VNP@ApDC in Miapaca-2 subcutaneous tumor model.** **(a)** The change of tumor volume over time with different treatments (n = 4). Data are presented as mean ± standard deviation of 4 independent experiments and analyzed using a two-way ANOVA, followed by Fisher's LSD multiple comparisons test. **(b)** Representative digital photograph of tumor tissues harvested from C57 mice on the day of sacrifice. **(c)** Tumor weight of different treatment groups on the day of sacrifice. **(d)** Fluctuation of body weight with different treatment (n = 4). **(e)** H&E stain images of heart, liver, spleen, lung, and kidney collected from mice after euthanasia. **(f)** Tumor sections were studied with H&E and IHC (anti-Ki67, anti-Caspase-3) stains. Scale bar: 100 μm.
